# Azithromycin resistance mutations in *Streptococcus pneumoniae* as revealed by a chemogenomic screen

**DOI:** 10.1099/mgen.0.000454

**Published:** 2020-10-19

**Authors:** Hélène Gingras, Kévin Patron, Philippe Leprohon, Marc Ouellette

**Affiliations:** ^1^​ Axe des Maladies Infectieuses et Immunitaires du Centre de Recherche du CHU de Québec and Département de Microbiologie, Infectiologie et Immunologie, Faculté de Médecine, Université Laval, Québec, Québec, Canada

**Keywords:** Azithromycin, chemical mutagenesis, Glutamine, macrolide, next generation sequencing, Resistance, *Streptococcus pneumoniae*

## Abstract

We report on the combination of chemical mutagenesis, azithromycin selection and next-generation sequencing (Mut-Seq) for the identification of small nucleotide variants that decrease the susceptibility of *Streptococcus pneumoniae* to the macrolide antibiotic azithromycin. Mutations in the 23S ribosomal RNA or in ribosomal proteins can confer resistance to macrolides and these were detected by Mut-Seq. By concentrating on recurrent variants, we could associate mutations in genes implicated in the metabolism of glutamine with decreased azithromycin susceptibility among *S. pneumoniae* mutants. Glutamine synthetase catalyses the transformation of glutamate and ammonium into glutamine and its chemical inhibition is shown to sensitize *S. pneumoniae* to antibiotics. A mutation affecting the ribosomal-binding site of a putative ribonuclease J2 is also shown to confer low-level resistance. Mut-Seq has the potential to reveal chromosomal changes enabling high resistance as well as novel events conferring more subtle phenotypes.

## Data Summary

The dataset supporting the conclusions of this article is available in the Sequencing Read Archive (https://www.ncbi.nlm.nih.gov/sra) repository under BioProject accession PRJNA636984, sample accessions SAMN15091567 to SAMN15091638.

**Impact Statement**

Antibiotic resistance is a major public health threat and strategies are needed for the identification of the target(s) of novel molecules and/or the mechanisms involved in their resistance. This article describes the coupling of chemical mutagenesis and next-generation sequencing for the genome-scale discovery of genes influencing the susceptibility of *Streptococcus pneumoniae* to azithromycin. Our approach allowed the identification of several genes previously reported in resistance but also of novel genes. This approach could be applied to other bacteria or phenotype to refine our understanding of the mode of action of antimicrobial molecules or of bacterial physiology.

## Introduction

*Streptococcus pneumoniae* is a versatile respiratory tract pathogen responsible for several diseases ranging from mild infections to life-threatening invasive pneumococcal diseases. Despite major progresses with vaccination, *S. pneumoniae* remains a significant cause of mortality and morbidity worldwide due to the increased contribution of non-vaccine serotypes. Some of these rising serotypes are also associated with antibiotic non-susceptibility, which on the long-term can obscure the benefits contributed by vaccination [1–3]. The rates of non-susceptible *S. pneumoniae* are high for penicillin, tetracycline, trimethoprim-sulfamethoxazole and macrolides [3–5].

Macrolides inhibit protein synthesis by binding to the peptide exit tunnel of the ribosome. The frequency of resistance to macrolides among pneumococci vary from 25–45 % depending on the geographic region [4]. Clinical resistance to macrolides in *S. pneumoniae* results from the acquisition by horizontal transfer of genes coding for the efflux pump Mef(E) or for the ribosomal RNA methylase ErmB [6]. Methylation by ErmB of 23S rRNA at nucleotide A2058 (* Escherichia coli* numbering) disrupts a key hydrogen bond between the antibiotic and its ribosomal target and confers high-level resistance including cross-resistance to macrolides, streptogramin B and lincosamides. In contrast, the Mef(E) efflux pump, which belongs to the major facilitator superfamily, confers lower resistance specifically to 14-membered and 15-membered macrolides. Resistance can also occur more rarely in the absence of *ermB* or *mef(E*) through mutations in the domain V of 23S rRNA [7–13] or in ribosomal proteins L4 or L22 [8, 12, 13]. Macrolides primarily interact with nucleotides A2058 and A2059 (* E. coli* numbering and this would correspond to A2060 and A2061 in *S. pneumoniae *) of the 23S rRNA and mutations at these positions are found in macrolide-resistant pathogens [14]. Ribosomal proteins mutations such as in L4 likely act indirectly by perturbing the conformation of rRNA [15].

A thorough understanding of the molecular adaptation of *S. pneumoniae* to macrolides could possibly reveal vulnerabilities that could be further exploited to subvert resistance. Indeed, subtle events coming either downstream of target inhibition or easing the acquisition of resistance can also be at play, as exemplified by the complex pool of mutations that can contextually improve gene expression and/or activate latent defence mechanisms in the presence of antibiotics [16]. Antibiotics act on bacterial metabolism, and antibiotic resistance involves changes in this metabolism [17]. Interventions that combine an antibiotic with an adjuvant, a molecule with no microbicidal activity but which potentiates the activity of the former by impacting on bacterial metabolism or physiology, may thus represent a strategy for reverting or preventing resistance [18].

In this study, we combined chemical mutagenesis with next-generation sequencing for the identification of genes or mutations influencing the susceptibility of *S. pneumoniae* to azithromycin (AZM). This strategy identified known mutations in 23S rRNA and in ribosomal protein L4 as important drivers of AZM resistance but also highlighted that mutations in genes pertaining to the metabolism of glutamine or rRNA maturation can alter susceptibility to AZM.

## Methods

### Growth conditions and molecular biology

*S. pneumoniae* R6 was grown at 35 °C with 5 % CO_2_ in either brain heart infusion (BHI) or C+Y medium [19] broth or on Trypticase soy agar with 5 % sheep blood (TSAII, Becton Dickinson). *S. pneumoniae* R6 culture under anaerobic conditions was performed in an anaerobic chamber. MICs were determined by microdilution in 96-well plates according to the CLSI guidelines in 0.1 ml cation-adjusted Müller–Hinton broth with 5 % lysed sheep blood and from at least three independent biological replicates. Point mutations often conferred a twofold increase in AZM MIC. This is considered to be within the experimental error of the technique but measurements were consistent between replicates and these MICs were conﬁrmed by macrodilution (performed in triplicates according to the CLSI guidelines) and were thus considered as genuine. AZM was purchased from Abcam. Genomic DNA (gDNA) was extracted using the Wizard Genomic DNA Puriﬁcation Kit (Promega). PCRs were performed using primers described in Table S1 (available in the online version of this article) using the Phusion enzyme (Thermo Scientific).

### Mut-Seq

*S. pneumoniae* Mut-Seq was performed as previously described [20]. A single *S. pneumoniae* R6 colony was grown in 100 ml of BHI until an OD at 600 nm of 0.2, and then the culture was split into 10 ml cultures. Ethyl methanesulfonate (EMS) (Sigma-Aldrich) was added at 8× or 16× its MIC. Cultures were incubated for 20 min at 35 °C before 10 ml of cold BHI medium was added. An 800 µl aliquot was incubated in 7.2 ml of BHI for 3 h for cell recovery. Bacteria were harvested by centrifugation (10 min at 4000 r.p.m.), resuspended in 200 µl of 1× PBS, and plated on casein tryptone (CAT) agar with 5 % (vol/vol) sheep blood plates containing 2 µg ml^−1^ of AZM. Non-mutagenized 10 ml cultures were similarly processed and used as a control. No clones were obtained from these controls upon AZM selection. Illumina Nextera XT sequencing libraries were prepared from gDNA derived from AZM-resistant clones according to the manufacturer’s instructions. The size distribution of Nextera XT libraries was validated using a 2100 Bioanalyzer and high-sensitivity DNA chips (Agilent Technologies). Sequencing was performed using an Illumina HiSeq2500 system (101-nucleotide paired-end sequencing) at a final concentration of 8 pM. Sequence reads were aligned to the *S. pneumoniae* R6 genome using the software bwa-mem [21]. The maximum number of mismatches was 4, the seed length was 32, and 2 mismatches were allowed within the seed. Read duplicates were marked using Picard (http://broadinstitute.github.io/picard), and we applied GATK for single-nucleotide polymorphism and indel discovery [22]. Several python and bash scripts were created to further analyse the data.

### DNA transformation for reconstruction of resistance

*S. pneumoniae* transformation was performed as previously described [23]. PCR fragments (5 kb) ampliﬁed from Mut-Seq mutants and covering the mutation of interest were transformed in *S. pneumoniae* R6 and selected on a series of CAT agar plates supplemented with 5 % (vol/vol) sheep blood and AZM (from 0.35 µg ml^−1^ to 2.0 µg ml^−1^). Mutations that allowed growth on higher AZM concentrations than the control were further validated by microdilution and Sanger sequencing. For whole-genome transformation (WGT), gDNA of a resistant clone was used instead of a PCR fragment for the transformation of *S. pneumoniae* R6, and the genome of clones with a higher MIC than mock transformed cells were sequenced on an Illumina MiSeq system.

### RNA extraction and RT-qPCR

RNA extraction and real-time quantitative PCR (RT-qPCR) were performed as described previously [24]. All RT-qPCR data were normalized according to the ampliﬁcation signals of *era* mRNA. The RT-qPCR primers are listed in Table S1.

### Growth curves

A 0.5 MacFarland bacterial suspension was made from *S. pneumoniae* grown overnight on TSAII plates. Cells were then diluted 1/400 in C+Y medium and distributed as 100 µl aliquots in a 96-well plate. Growth (OD_600 nm_) was monitored every 30 min using a Cytation 5 multimode reader (BioTek). After 6 h of growth (OD_600 nm_ of 0.02), 100 µl of the antibiotics at the selected concentration was added to the plate. Data analysis was completed using the Gen5 software and reported with GraphPad Prism.

### Growth inhibition curves

The MIC of the GlnA-specific inhibitor l-methionine sulfoximine (MSO) (Sigma) was determined using microdilution in 96-well plates according to CLSI guidelines. The last concentration without any growth defect (i.e. 4 µg ml^−1^) was used for the drug-inhibition assays with AZM, tetracycline (Sigma) and ciprofloxacin (Sigma). Then, 96-well plates were seeded with *S. pneumoniae* R6 as described above in the presence of antibiotic with and without MSO. Growth (OD_600 nm_) was monitored after 20 h of incubation at 35 °C in a Cytation 5 multimode reader. For each drug concentration, relative growth was estimated as the percentage of growth compared to untreated control.

## Results

*S. pneumoniae* R6 was treated with EMS at a concentration equivalent to 8× or 16× its MIC. The mutagenized population was seeded onto nutrient agar plates under aerobic (duplicate 1) and anaerobic (duplicate 2) atmosphere that were supplemented with AZM at a concentration that is lethal to a non-mutagenized control population. Direct selection on solid agar prevented the most resistant clones from outcompeting the ones with a more subtle phenotype. We could also assess the impact of oxygen levels on AZM resistance as *S. pneumoniae* is a catalase-negative facultative anaerobe for which oxygen concentration was shown to influence metabolism [25]. AZM selection with 2 µg ml^−1^ was optimal in yielding the highest number of clones for aerobic and anaerobic conditions and no growth for control cells. Resistant mutants were picked from the plates and their AZM MIC independently confirmed. Mutants had varying levels of resistance to AZM, the ones obtained under aerobic condition often having a stronger resistance phenotype (Table 1). None of the mutant selected under anaerobic conditions had a high level of resistance (Table 1). The genomes of 26 and 44 mutants respectively selected under aerobic and anaerobic atmosphere were sequenced and compared to the genome of the parental wild-type (WT) clone.

**Table 1.** Resistance levels of AZM-resistant mutants selected under aerobic and anaerobic atmosphere

Mutants selected under aerobic atmosphere				Mutants selected under anaerobic atmosphere			
		Mutations				Mutations	
Strain or mutant name	AZM MIC (µg ml^−1^)*	50S ribosomal protein L4†	23S ribosomal RNA‡	Strain or mutant name	AZM MIC (µg ml^−1^)*	50S ribosomal protein L4†	23S ribosomal RNA‡
R6 WT	0.25	–	–	R6 WT	0.25	–	–
M1§	1024	–	A2060T (4)	M27||	1	G71R	–
M2§	1024	–	A2060T (4)	M28||	1	K68E	–
M3§	1024	–	A2060T (4)	M29**§**	0.5	–	C2613T (2)
M4§	64	–	A2061C (4)	M30||	1	K68E	–
**M5¶**	1	G71R	–	**M31¶**	0.5	–	–
**M6¶**	1	G71R	–	M32**§**	0.5	–	C2613T (2)
M7	1	A189ACCATGG	–	M33||	1	K68E	–
**M8¶**	1	G71R	–	**M34¶**	0.5	–	–
M9§	512	–	A2061G (3)	**M35¶**	1	–	C2613T (1)
M10§	1024	–	A2060T (4)	M36||	1	K68E	–
M11§	1024	–	A2060T (4)	M37¶	0.5	–	–
M12§	64	–	A2061C (4)	M38§	0.5	–	C2613T (1)
M13§	1024	–	A2060T (4)	**M39¶**	0.5	–	–
**M14¶**	1	G71R	–	M41**§**	0.5		C2613T (2)
**M15¶**	1	G71R	–	M42||	1	G71R	–
**M16¶**	1	G71R	–	M43§	0.5	–	C2613T (1)
**M17¶**	1	G71R	–	M44||	0.5	K68E	–
**M18¶**	1	Q67R	–	M45||	1	K68E	–
M19||	0.5	G71R	–	**M46¶**	1	–	C2613T (1)
**M20¶**	1	G71R	–	M47||	1	K68E	–
**M21¶**	1	G71R	–	M48||	1	K68E	–
M22§	2	–	C2613T (4)	M49**§**	0.5	–	A2061G (1)
M23§	2	–	C2613T (4)	M50**§**	2	–	C2613T (4)
**M24¶**	1	Q67R	–	M51**§**	1	–	C2613T (4)
**M25¶**	1	G71R	–	M52**§**	0.5	–	C2613T (4)
M26§	1024	–	A2060T (4)	**M53¶**	1	–	C2613T (1)
				M54**§**	1	–	C2613T (3)
				M55**§**	0.5	–	C2613T (2)
				M56||	1	K68E	–
				M57**§**	1	–	C2613T (4)
				M58§	0.5	–	C2613T (1)
				M59||	0.5	K68E	–
				M60**§**	1	–	C2613T (3)
				M61**§**	1	–	C2613T (3)
				M62**§**	0.5	–	C2613T (2)
				M63**§**	0.5	–	C2613T (2)
				M64||	1	G71R	–
				M65||	1	G71R	–
				M66**§**	2	G71R	C2613T (4)
				M67**§**	0.5	–	C2613T (4)
				**M68¶**	2	–	C2613T (1)
				M69**§**	1	–	C2613T (4)
				M71||	1	G71R	–
				M72||	1	G71R	–
				M73||	1	G71R	–

*MIC measurements were replicated for a minimum of three times.

†The amino acid substitution and position in the protein is indicated, except for the small insertion in mutant M7 for which the position indicated refers to the nucleotide position in the gene.

‡Indicated is the mutation in nucleotide and position in the gene. The number of mutated gene copies is indicated within parentheses.

§The AZM MIC of these mutants is fully explained by mutations in 23S rRNA.

||The AZM MIC of these mutants is fully explained by mutations in ribosomal protein L4.

¶The AZM MIC of these mutants is expected to involve additional mutations than those in 23S rRNA or ribosomal protein L4. Also, in bold.

A total of 162 single-nucleotide variants (SNVs) were identified in the 26 AZM-resistant mutants selected under aerobic condition, with 124 SNVs in 89 coding sequences (Table S2) and 38 SNVs in intergenic regions (Table S3). For the 44 mutants selected under anaerobic condition, 431 and 52 SNVs were observed in coding and intergenic sequences, respectively (Tables S2 and S3). We concentrated on recurrent SNVs that were present in independent mutants. The two genes most frequently mutated, as expected from the mode of action of AZM, were the one coding for the 50S ribosomal protein L4 and one or several of the four gene copies of the 23S ribosomal RNA (rRNA) (Table 1). Four different 23S rRNA mutations were observed. The A2060T and A2061G/C mutations prevailed among mutants derived from aerobic conditions while the C2613T mutation was over-represented among those selected in the absence of oxygen (Table 1). In general, the levels of AZM resistance among the mutants correlated with the nature and number of 23S rRNA mutations (Table 1). This correlation was tested by transformation of the mutations into *S. pneumoniae* R6. The A2060T and A2061G mutations produced the most resistance to AZM and the level of resistance of the transformants was proportional to the number of mutated copies (Table 2). Twelve aerobic mutants out of 26 had mutations in 23S rRNA. These mutations were necessary for high-level resistance and sufficient to explain the AZM resistance levels in these mutants (Table 1). Only a modest decrease in susceptibility was found in cells selected under anaerobic conditions, with 24 mutants having mutations in the 23S rRNA (23 of which had the C2613T mutation). Mutations in the 23S rRNA could explain resistance levels for the majority of these mutants selected under anaerobic conditions with the exception of seven (Table 1), suggesting the presence of additional resistance mechanisms in the latter.

**Table 2.** Contribution of 23S rRNA mutations in resistance to AZM

Strain	23S rRNA mutation	AZM MIC (µg ml^−1^) aerobic/anaerobic*			
		No. of mutated gene copies			
		1	2	3	4
* S. pneumoniae* R6	C2613T	0.25/0.5	0.5/0.5	1/1	2/2
	A2060T	0.5/0.25	1024/512	nd/nd	nd/nd
	A2061G	0.5/0.5	1024/512	nd/nd	nd/nd
	A2061C	0.5/0.25	32/nd	64/nd	nd/nd

*nd, Not done because the AZM MIC of the most resistant mutant harbouring the mutation was already reached. MICs were performed independently for a minimum of three times.

Most mutants with no mutations in the 23S rRNA genes had one of three non-synonymous mutations in the gene coding for the 50S ribosomal protein L4, in addition to one frameshift mutation (Table 1). These were all located within a highly conserved region of *S. pneumoniae* L4 (_63_KPWRQKGTGRAR_74_). Mutations in L4 occurred in mutants with the lowest MICs to AZM (Table 1). The G71R amino acid substitution was the most prevalent among aerobic mutants and also represented roughly half of L4 mutations detected in anaerobic clones (Table 1). Surprisingly, the mutation K68E was also abundant but exclusively found in cells selected under anaerobic conditions. Consistent with the MIC found in mutants, transformation of the three non-synonymous mutations (G71R, K68E and Q67R) into *S. pneumoniae* R6 conferred a twofold increase in AZM MIC (Table 3). In the absence of oxygen the increase in AZM MIC was fourfold (Table 3). Ribosomal protein L4 variants were thus sufficient to explain the AZM resistance of every anaerobic mutant harbouring such a variant, while additional mutations were likely involved for mutants selected under aerobic atmosphere, except for M19 (Table 1). Mutant M34, with no mutation in either L4 or 23S rRNA, had a mutation in ribosomal protein L22 (Table S2), another known contributor of resistance to macrolides [7, 9, 14], but we did not specifically assess its contribution to the phenotype of this mutant.

**Table 3.** Contribution to AZM resistance of mutations in 50S ribosomal protein L4 under aerobic and anaerobic atmosphere

Strain	spr0189 mutation*	AZM MIC (µg ml^−1^)†	
		Aerobic	Anaerobic
* S. pneumoniae* R6	None	0.25	0.25
	Q67R	0.5	1.0
	K68E	0.5	1.0
	G71R	0.5	1.0

*spr0189, 50S ribosomal protein L4. Indicated is the amino acid substitution and position in the protein.

†MICs were performed in triplicates with identical values.

**Fig. 1.**
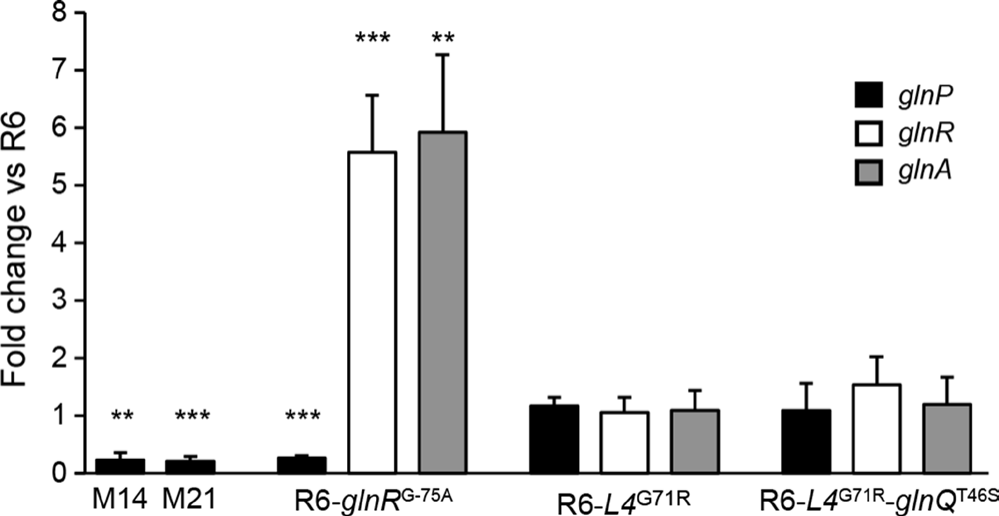
Impact of mutations on gene expression. Fold change in expression of *glnP* (black bars), *glnR* (white bars) and *glnA* (grey bars) in mutants M14 and M21 or in *S. pneumoniae* R6 transformants harbouring a mutation upstream of spr0443 (glnR; from M6), a mutation in spr0189 (ribosomal protein L4^G71R^) alone or in combination with a mutation in spr1121 (GlnQ^T46S^). Expression changes are relative to R6 wild-type. **P ≤0.01; ***P ≤0.001.

Outside L4 and 23S rRNA we investigated additional gene candidates by prioritizing genes mutated in several mutants, as this recurrence could be linked with the resistance phenotype. Noticeable among mutants whose AZM resistance could not be solely explained by mutations in ribosomal protein L4 were mutations in the coding or regulatory regions of genes pertaining to glutamine metabolism. Six different non-synonymous mutations were found in genes spr1120 (*glnP*, two mutations) and spr1121 (*glnQ*, four mutations) respectively coding for the membrane-spanning and ATP-binding domains of the ATP-binding cassette (ABC) transporter GlnPQ (Table 4). This transporter, which is the main uptake system for glutamine in *S. pneumoniae* [26, 27], displayed the highest prevalence and diversity of mutations (Table S2). Each of the spr1120-21 non-synonymous mutations increased the AZM MIC by twofold when independently transformed into *S. pneumoniae* R6 harbouring also a mutation in L4 (Table 4). Interestingly, a deletion of one nucleotide in the −35 element of the predicted promoter for spr1120 (*glnP*) was detected in mutants M14 and M21 (Table 4, Fig. S1a). From its location the mutation is likely to influence the transcription of the gene and the expression of *glnP* (spr1120) was indeed found to be decreased in both mutants (Fig. 1). This result is in contrast to the coding *glnPQ* mutations or to mutations in ribosomal protein L4, which had no impact on the expression of the gene (Fig. 1). Besides *glnPQ*, a G to A transition upstream of the gene spr0443 coding for the transcriptional regulator GlnR was detected in mutant M6 (Table 4). GlnR mediates the repression of genes involved in glutamine synthesis (*glnA*) and uptake (*glnPQ*), in addition to the repression of its own expression [27]. The mutation increased AZM MIC by twofold when transformed in the presence of L4 mutations in *S. pneumoniae* R6 (Table 4). It is salient to point out that the insertion of the mutation in a wild-type R6 background was not associated with a change in AZM susceptibility. The mutation is localized within the GlnR-binding site upstream of the *glnRA* operon (Fig. S1b) and is likely preventing GlnR binding to its operator. This hypothesis was supported by the observation that the expression of *glnR* and *glnA* (coding for the glutamine synthetase) is derepressed in the presence of the mutation (Fig. 1). Mutations in ribosomal protein L4 or in the coding region of *glnPQ* used as control did not influence the expression of *glnRA* (Fig. 1). As expected from the regulon of GlnR, the genes *glnPQ* were found to be downregulated due to the increased expression of GlnR in the presence of the mutation (Fig. 1). Overall, mutations in genes pertaining to glutamine metabolism were thus additive with those in ribosomal protein L4 to fully reconstruct the AZM resistance of mutants M5, M6, M14, M15, M18, M19, M21, M24 and M25 (Table 4).

**Table 4.** Contribution to AZM resistance of mutations in genes pertaining to glutamine metabolism and other recurrent mutations

Strain	AZM MIC (µg ml^−1^)*	Mutations (AZM MIC µg ml^−1^)†					
		spr0189 (L4)	spr1120 (*glnP*)‡	spr1121 (*glnQ*)‡	spr0443 (*glnR*)‡	spr0538‡	spr1811‡
R6	0.25						
M5§	1.0	G71R (0.5)		N91S (1.0)			
M6§	1.0	G71R (0.5)			G-75A|| (1.0)		
M14	1.0	G71R (0.5)	CT-54C||				
M15§	1.0	G71R (0.5)	M651I (1.0)				
M16§	1.0	G71R (0.5)				G-5C|| (1.0)	
M17§	1.0	G71R (0.5)					Q134* (1.0)
M18	1.0	Q67R (0.5)		V179I (1.0)			
M19	0.5	G71R (0.5)		H233D (1.0)			
M21	1.0	G71R (0.5)	CT-54C||				
M24	1.0	Q67R (0.5)	A619D (1.0)				
M25	1.0	G71R (0.5)		T46S (1.0)			

*The AZM MIC of the strain or mutant under aerobic atmosphere. All measurements were repeated a minimum of three times.

†Unless indicated otherwise, represent changes in amino acid and positions in the protein. An asterisk denotes a non-sense mutation. The AZM MIC indicated within parentheses are those of *S. pneumoniae* R6 transformed with the appropriate mutation.

‡Mutations in these genes were transformed into *S. pneumoniae* R6 harbouring G71R or Q67R mutations in ribosomal protein L4 (MIC AZM 0.5 µg ml^−1^). The AZM MICs indicated (within parenthesis) thus represent the sum of the contribution of the mutations along with the one in L4 mutations.

§The MICs of penicillin (0.0156 µg ml^−1^) and ceftriaxone (0.03 µg ml^−1^) for these transformants were equal to those of *S. pneumoniae* R6 wild-type or of *S. pneumoniae* R6 harbouring the G71R mutation in ribosomal protein L4.

||These represent intergenic mutations expressed in nucleotides. The position indicated corresponds to the nucleotide position upstream of the start codon.

**Fig. 2.**
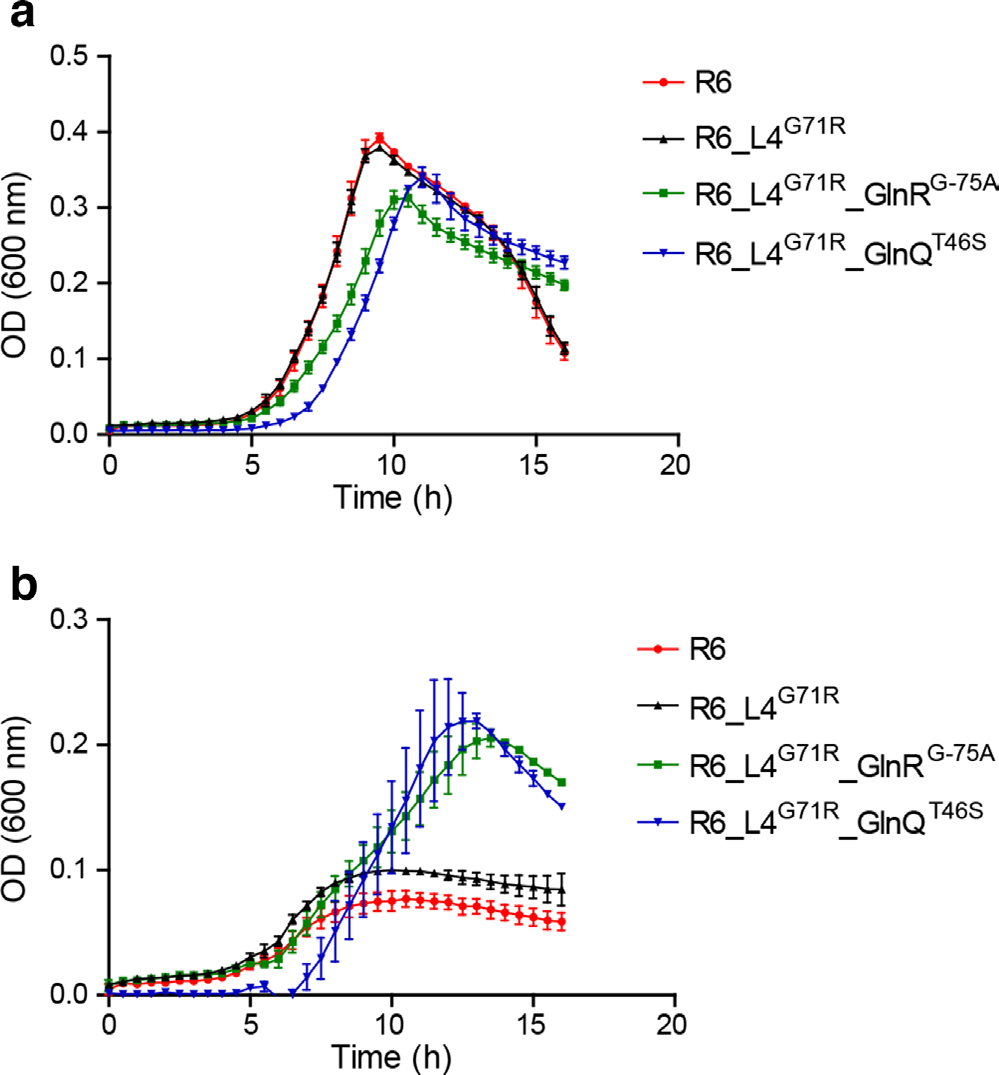
Impact of mutations on growth. Growth of *S. pneumoniae* harbouring different sets of mutations detected by Mut-Seq in the absence of AZM (a) or in the presence of 0.125 µM AZM (b) For R6 transformants, the gene affected by the mutation is indicated in superscript. R6, *S. pneumoniae* R6 wild-type.

**Fig. 3.**
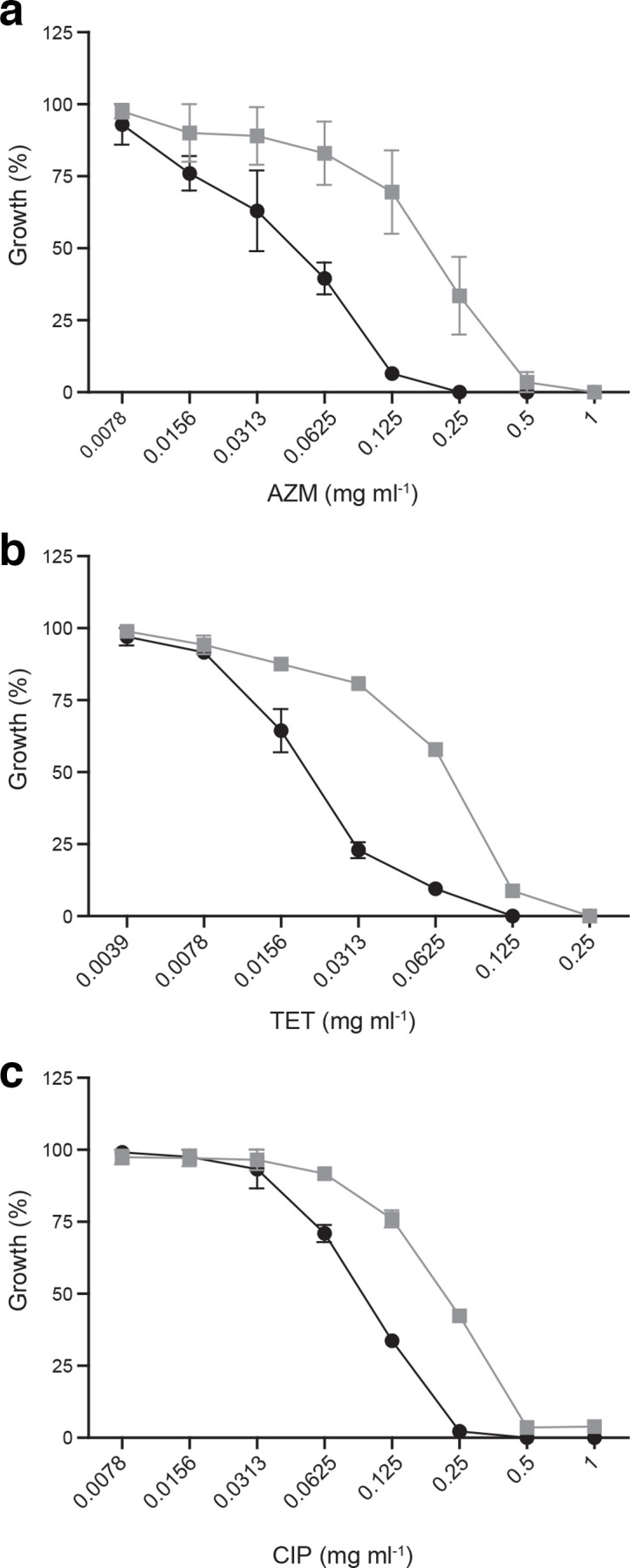
The inhibition of glutamine synthetase sensitizes *S. pneumoniae* to different antibiotics. *S. pneumoniae* R6 wild-type growth inhibition curves of AZM (a) tetracycline (b) and ciprofloxacin (c) in the absence (grey) or presence (black) of the GlnA inhibitor MSO at 4 µM. Data is expressed as the relative growth compared to non-treated R6 wild-type after overnight incubation.

In contrast to the G71R mutation in L4, the mutations in GlnQ (from M25) or upstream of *glnR* (from M6) were slightly detrimental to growth in the absence of AZM (Fig. 2a) but conferred an advantage in the presence of AZM (Fig. 2b). The activity of GlnA is tightly regulated, being feed-back inhibited by intracellular glutamine [27], and it is probable that the decreased glutamine uptake translates into higher GlnA activity in the AZM-resistant mutants. We investigated this idea further by inhibiting the glutamine synthetase GlnA, the main glutamine biosynthesis enzyme, and tested whether this would enhance AZM lethality. The GlnA-specific inhibitor MSO [28–30] increased the susceptibility of *S. pneumoniae* to AZM by fourfold (Fig. 3a). MSO was not toxic to *S. pneumoniae* at the concentration used (Fig. S2). This potentiation was not specific to AZM however and also occurred in the case of tetracycline (another translation inhibitor) (Fig. 3b) and ciprofloxacin (Fig. 3c), a DNA replication inhibitor for which a link with glutamine had previously been observed [24].

Similarly to the mutants described above, the AZM resistance phenotype of mutants M8, M16, M17 and M20 likely involved additional gene candidates beside ribosomal protein L4 but these did not have mutations in genes related to glutamine or other recurrent genes. For these mutants we used WGT [31] to discriminate mutations contributing to AZM resistance from bystander mutations. This approach involves the transformation of gDNA derived from the mutants into *S. pneumoniae* R6 expressing the G71R variant of ribosomal protein L4 (AZM MIC 0.5 µg ml^−1^) and selecting for transformants displaying an increased resistance to AZM. Mutant M18 harbouring a V179I mutation in GlnQ was used as a positive control. For the four mutants, AZM-resistant transformants were selected in a single step and no spontaneous AZM-resistant mutants were obtained from the DNA-free control plates. In all cases, three transformants were chosen at random for further study by NGS. All transformants derived from M18 had an AZM MIC of 1 µg ml^−1^ and acquired the V179I mutation in GlnQ. This validated the approach, in addition to confirming the role of GlnPQ mutations in increasing AZM MIC. Transformants derived from M16 had an AZM MIC of 1 µg ml^−1^ and acquired a single G to C transition located five nucleotides upstream of the initiation codon of spr0538 coding for ribonuclease J2 (RNase J2). This mutation derived from M16 (Table S3) was transformed into *S. pneumoniae* R6, which increased the AZM MIC by twofold (Table 4). Transformants derived from M17 all acquired from the mutant a non-sense mutation in spr1811, truncating this hypothetical protein at glutamine 134 (Tables 4 and S2). The mutation increased AZM MIC by twofold when introduced into *S. pneumoniae* R6 (Table 4). WGT did not allow additional AZM resistance genes for M8 and M20 to be pinpointed.

## Discussion

In this study, we used Mut-Seq to search for genes for which a change in sequence or expression (through mutation of regulatory regions) alter the susceptibility of *S. pneumoniae* to the macrolide antibiotic AZM. Mass administration of AZM is a cornerstone in the control of trachoma [32, 33] with additional benefits against overall childhood mortality [34]. Azithromycin has also anti-inflammatory activity, which also contributes to improved health outcomes (reviewed in [35]). This success has a price, however, since the massive use of AZM increased the microbial resistance gene pools against macrolides [36]. A metanalysis confirmed increased resistance to macrolides in *S. pneumoniae* in areas using AZM mass administration, although further work is required to establish for how long this resistance is maintained and its impact in treatment outcomes (reviewed in [37]). This increase in resistance needs to be closely monitored for not losing the benefits of AZM for both trachoma control and for its dual antibacterial anti-inflammatory activity helpful against a diversity of life-threatening infections.

Resistance to AZM in *S. pneumoniae* predominantly involves target modification and drug efflux from the acquisition of the genes *erm(B*) and *mef(E*) by horizontal transfer, respectively. However, some resistant isolates can be negative for *erm(B*) and *mef(E*) and have chromosomal mutations in 23S rRNA genes or in the genes coding for ribosomal proteins L4 or L22 [8, 9, 12, 13, 38, 39]. Mutations in 23S rRNA near to the macrolide binding nucleotide A2060 (equivalent to A2058 in *E. coli *) are known to confer high-level macrolide resistance [14]. Ribosomal proteins L4 and L22 have domains that extend near the macrolide-binding site into the peptide exit tunnel [40] and mutations affecting these highly conserved domains are likely preventing macrolide binding by perturbing the conformation of rRNA. Most of our mutants had mutations in either 23S rRNA or in ribosomal protein L4 but never in both (except for M66). The level of AZM resistance depended on the nature and position of the mutations in the 23S rRNA and was also proportional to the number of mutated alleles, as previously described [7, 9–12]. Mutants raised under aerobic conditions often had more 23S rRNA alleles mutated than their counterparts raised anaerobically. The latter also had fewer mutations affecting nucleotides 2060 and 2061, which are the most potent sites for high-level resistance. Mutations in rRNA confer a fitness cost [41] and it is possible that mutations at these positions are more detrimental under anoxic conditions. Strict anaerobic conditions are associated with higher growth and biomass for *S. pneumoniae* R6 [42], implying there might be a greater demand for the translation machinery.

Common to several *S. pneumoniae* mutants were mutations in genes involved in the metabolism of glutamine. Glutamine had previously been shown to influence the susceptibility of *S. pneumoniae* and *Staphylococcus aureus* towards cell-wall-synthesis inhibitors [24, 43]; the inhibition of GlnA even allowing partial re-sensitization of penicillin-resistant *S. pneumoniae* [24]. In *Helicobacter pylori *, a mutant selected *in vitro* for resistance to the macrolide antibiotic clarithromycin was shown to harbour a mutation in a gene coding for the glutamine ABC transporter [44], the orthologue of the gene observed here for several of our AZM-resistant mutants. The authors did not experimentally evaluate the role of the mutation in clarithromycin susceptibility but given our data it is likely to contribute. Most of the mutations detected in our mutants indeed affected GlnPQ, the main glutamine transporter in *S. pneumoniae* [26, 27], either in a direct fashion (i.e. mutations in the coding sequence) or indirectly by influencing its expression and each mutant class increased the AZM MIC. In addition to being a building block for protein synthesis, glutamine is required for the biosynthesis of nitrogen-containing compounds and its synthesis by GlnA is the primary reaction for the assimilation of ammonia; GlnA catalyses the ATP-dependent conversion of l-glutamate and ammonia to l-glutamine. The expression of GlnA is tightly regulated, it is high under glutamine limitation and decreases upon glutamine supplementation [27]. This regulation is lost in the absence of the transcriptional regulator GlnR [27] or upon mutation of its operator, as observed here for mutant M6 with an increased *glnA* expression. The activity of GlnA is also feed-back inhibited in the presence of glutamine [27]. The decreased glutamine uptake expected from the downregulation or mutation of *glnPQ* is thus likely to increase the activity of GlnA. In *E. coli *, high levels of ammonia in the medium were shown to aggravate the toxicity of tetracycline by a mechanism that remains to be clarified [45]. We could hypothesize that increased GlnA activity, and hence higher ammonia assimilation, may protect against the action of antibiotics. In *Mycobacterium tuberculosis *, the inhibition of GlnA by MSO was shown to enhance the lethality of bedaquiline [46]. Interestingly, supplementation with glutamine alone (in the absence of MSO) did not protect against bedaquiline action, suggesting that the activity of GlnA per se is predominant over glutamine concentrations in mediating the phenotype [46]. Given the likely increase in GlnA activity in our mutants, the same may be happening here with the assimilation of ammonia by GlnA being more important than the concentration of its product. The elucidation of the exact mechanisms at play will require further work. It is possible that GlnA activity may have a non-specific protective effect against antibiotics, since glutamine metabolism was shown to alter susceptibilities to many antibiotics [24, 43, 46].

A mutation targeting the last nucleotide of a Shine–Dalgarno-like motif upstream of gene spr0538 increased the AZM MIC of mutant M16 by twofold. This gene codes for the RNase J2. Ribonuclease J are RNases with 5′−3′ exonuclease activity (and possibly endonuclease activity) that are involved in the maturation of 16S rRNA [47, 48]. RNase J2 interacts with RNase J1 and appears to regulate its activity. The impact of the mutation on the expression of RNase J2 has not been tested but it is noteworthy that in *E. coli* and *Salmonella enterica* serovar Typhimurium, the presence of an extra three to eight nucleotides at the 5′ end of 16S rRNA as a result of incomplete processing by RNase G is sufficient to induce low-level resistance to aminoglycosides [49].

By transfecting back mutations recurring in the same genes or mutations retrieved by WGT, we could reconstruct the MIC of all but two (M8 and M20) aerobic mutants and of all but seven (M35, M38, M43, M46, M53, M58, M68) anaerobic mutants. For the latter we did not try WGT. The remaining mutations involved in resistance are likely mutant specific (Tables S2 and S3). Mut-Seq has the potential to reveal chromosomal changes enabling high resistance as well as additional novel events conferring more subtle phenotypes.

## Supplementary Data

Supplementary material 1
