# Supplementary material for: Azithromycin resistance mutations in Streptococcus pneumoniae as revealed by a chemogenomic screen
Source: Microb Genom. 2020 Oct 19;6(11):mgen000454. doi: 10.1099/mgen.0.000454 (PMC7725334; doi:10.1099/mgen.0.000454)
Supplement: Supplementary material 1 [file mgen-6-454-s001.pdf]

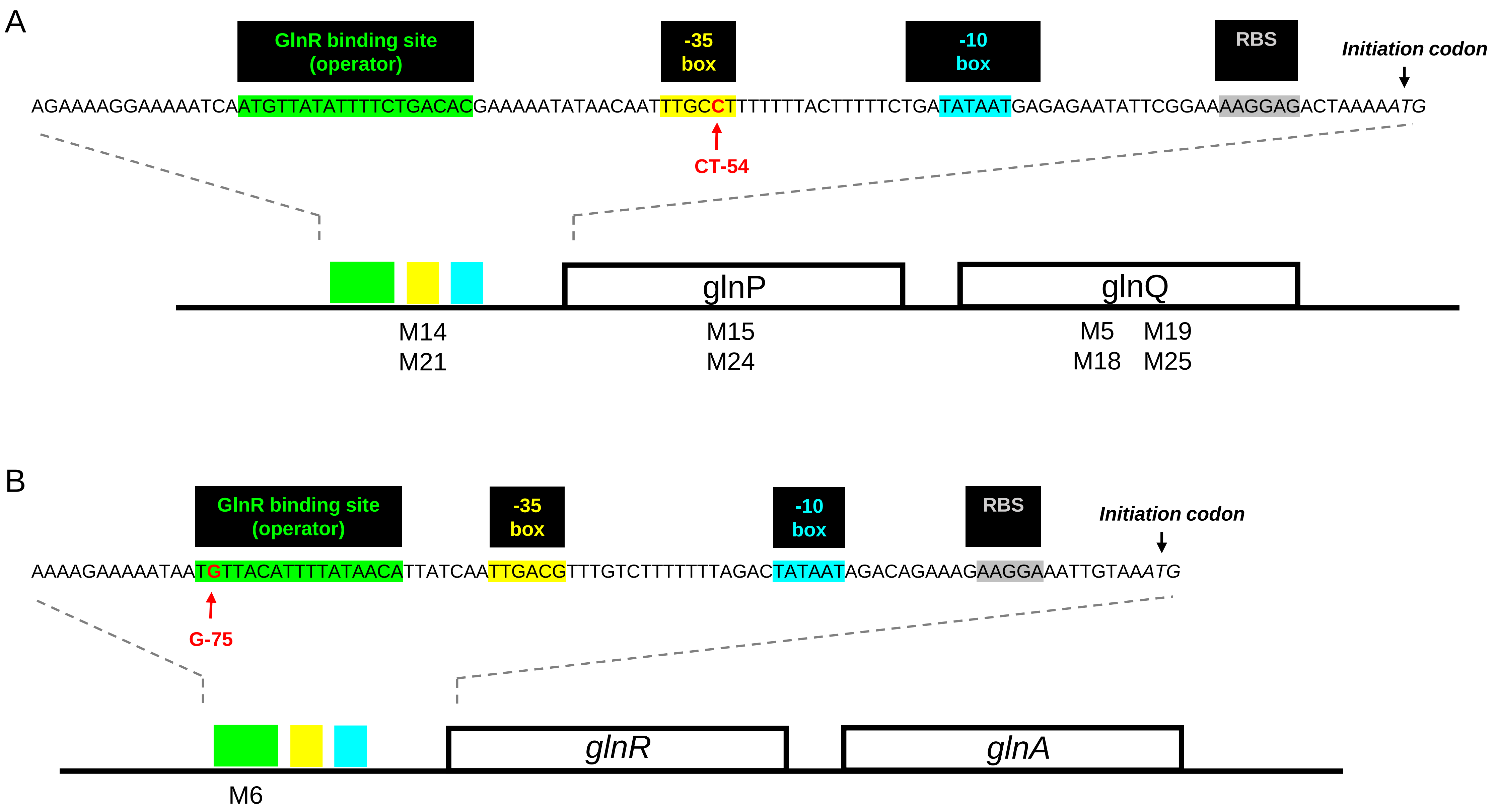

A
GlnR binding site (operator)
-35 box
-10 box
RBS
Initiation codon
AGAAAAGGAAAAATCAATGTTATATTTTCTGACACGAAAAATATAACAATTTGCCTTTTTTTACTTTTTCTGATATAATGAGAGAATATTCGGAAAAGGAGACTAAAAATG
CT-54
glnP
glnQ
M14
M21
M15
M24
M5
M19
M18
M25
B
GlnR binding site (operator)
-35 box
-10 box
RBS
Initiation codon
AAAAGAAAAATAATGTTACATTTTATAACATTATCAATTGACGTTTGTCTTTTTTTAGACTATAATAGACAGAAAGAAGGAAATTGTAAATG
G-75
glnR
glnA
M6

Figure S1. Summary of the mutational and expression status for the *glnPQ* and *glnRA* operons. Schematics of the glnPQ (**A**) and glnRA (**B**) operons with their coding and regulatory regions. Details about the regulatory regions are shown above the genes. Mutations detected in the regulatory regions (see Table 4) are indicated in red. The name of the mutants harboring mutations are indicated below the appropriate coding or regulatory regions. Mutation in the -35 box of the *glnPQ* operon, as shown in M6 decreases its own expression (Fig. 1). The mutation in the GlnR binding site of the *glnRA* operon leads to increased expression of its own operon and to decreased expression of the glnPQ operon (Fig. 1).

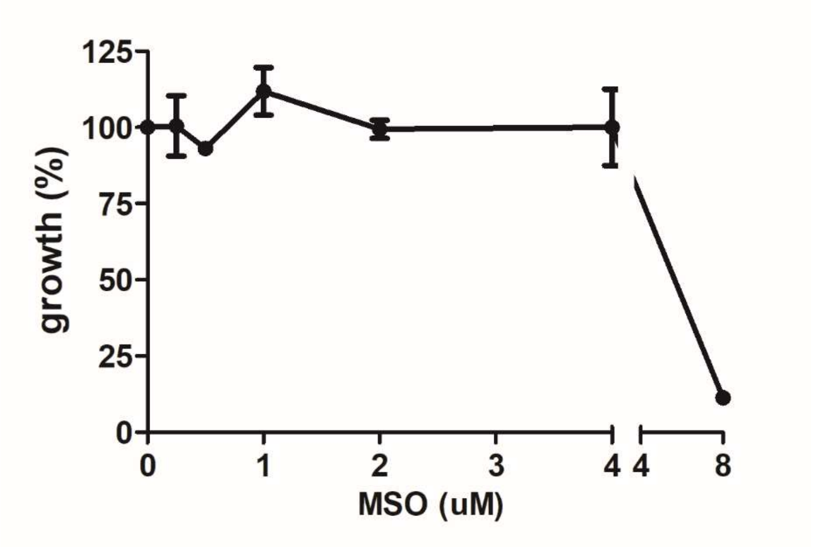

Figure S2. Assessment of MSO toxicity. Growth of *S. pneumoniae* R6 wild-type in the presence of increasing concentrations of MSO. Data is expressed as the relative growth compared to non-treated R6 wild-type after overnight incubation.

Table S1. Primers used in this study.

**Primers used for Mut-Seq**			
short-spr0189-F	AAACCTTGCAGGACGTATGG	short -spr0189-R	TGCTTCAAGTTGAGCCATTG
5kb-spr0189-F	CGGATGAGGAGACCGAATTA	5kb-spr0189-R	ATTTTTCCACGGAACTCACG
short-spr1121-F	TGAAATCGTTCGTGGTGGTA	short-spr1121-R	ATCTGCCAAATCAAGGATGG
5kb-spr1121-F	ATGGCAGGGATGACAAAGAC	5kb-spr1121-R	TGATACTGCTGGTCGTCTGC
short-spr1120-F	TGCCATTGTCATCGGAATTA	short-spr1120-R	TATTTTCACGGACGTGGTCA
4kb-spr1120-F	GTTCCTGACGGTTGACCACT	4kb-spr1120-R	CTTGCGTTTAGCCAGGTCTC
5kb-spr0443-F	ATGCTAGTTGGGGAACAACG	5kb-spr0443-R	TATCCTCACCCACTCCCAAG
Inside-spr0443-F	TGTTCCAATCTGCACCATGT		
5kb-spr0538-F	TGCTTTCAAGTTGGCTTGTG	5kb-spr0538-R	AGGCTGGACTTCCCAGATTT
Inside-spr0538-F	CTGGGTACGGTGCAAGAAAT		
5kb-spr1811-F	GTTTCAAGCGAGCTTTTTGG	5kb-spr1811-R	TAGGGCCAGACAAGACCATC
Inside-spr1811-F	CACAAAAGGGGAGATTGGAA		
sprr-common-F	CCGAAGCTGTGGATACCTTT		
sprr02-unique-R	CTAAACCTCTTAACTCAATTACTCCGCCAGT		
sprr05-unique-R	AGCCGAACATCAATTTGGAA		
sprr08-unique-R	AAGAGAGAACCAAACTGATTCCCT		
sprr11-unique-R	CAAGTCAAAAACTTGGTACAAAGAAC		
Mutall975-F	TCAGACTGCGAGTGCTAAGATCCG	Mutall3085-R	CTTCCCTATCTCACAGGGGGCAACC
Mut2613CT-F	GAGACAGTTCGGTCTCTATCCGTCGCGGGC	Mut2613CT-R	GCCCGCGACGGATAGAGACCGAACTGTCTC
Mut2061AG-F	CCGCGACAGGACGGAGAGACCCCATGGAG	Mut2061AG-Rv	CTCCATGGGGTCTCTCCGTCCTGTCGCGG
Mut2061AC-F	CCGCGACAGGACGGACAGACCCCATGGAG	Mut2061AC-Rv	CTCCATGGGGTCTGTCCGTCCTGTCGCGG
Mut2060AT-F	CCGCGACAGGACGGTAAGACCCCATGGAG	Mut2060AT-Rv	CTCCATGGGGTCTTACCGTCCTGTCGCGG
**Primers used for RT-qPCR**			
era-F	GTAGTATGGCCCGTCGTGAT	era-R	TAGCTTTTTATCGCGCCAGT
spr0443-F	CATGGATCGTCTGCTTGAAA	spr0443-R	CTTGGATTTCGCTTCACGTT
spr0444-F	TCTTGAGTTGCGTTCAGTGG	spr0444-R	TTCGATAGGAGCTGGTGCTT
spr1120-F	CTGGGGAATTCCAAACTTCA	spr1120-R	ATTGAGTGAGAGGGCAATGG
spr1121-F	CTCGTGGCCTAGCAATGAAT	spr1121-R	ACGTCTCCAACCATCTCAGG

Table S2. Non-synonymous mutations detected in the coding regions AZM-resistant mutants. Genes with mutations in more than one mutant are highlighted with alternating yellow and grey shading.

Mutant name	Gene name	Position in gene	WT allele	Alt allele	WT amino acid	Alt amino acid	Function
M24	spr0003	73	G	A	A	T	Hypothetical protein
M59	spr0010	229	G	A	A	T	Conserved hypothetical protein
M52	spr0029	79	C	T	D	N	Degenerate transposase orf1
M66	spr0037	859	G	A	G	S	Involved in fatty acid phospholipid synthesis
M67	spr0037	859	G	A	G	S	Involved in fatty acid phospholipid synthesis
M69	spr0037	859	G	A	G	S	Involved in fatty acid phospholipid synthesis
M37	spr0042	412	G	A	L	F	Transposase orf1
M7	spr0043	34	G	A	V	M	Transport ATP-binding protein ComA
M51	spr0051	1114	G	A	E	K	Phosphoribosylaminoimidazolecarboxamide formyltransferase
M59	spr0052	572	G	A	G	E	Phosphoribosylglycinamide synthetase
M60	spr0057	2119	C	T	D	N	Beta-N-acetyl-hexosaminidase precursor
M63	spr0057	2119	C	T	D	N	Beta-N-acetyl-hexosaminidase precursor
M12	spr0059	1362	C	G	N	K	Beta-galactosidase 3
M59	spr0064	517	G	A	A	T	Tagatose-6-phosphate ketose aldose isomerase
M18	spr0065	197	G	A	G	D	Aldose-1-epimerase mutarotase
M66	spr0066	671	C	T	A	V	Conserved hypothetical protein
M67	spr0066	671	C	T	A	V	Conserved hypothetical protein
M69	spr0066	671	C	T	A	V	Conserved hypothetical protein
M19	spr0070	240	G	A	W	*	Trk transporter membrane-spanning protein
M39	spr0073	561	C	A	R	S	Hypothetical protein
M55	spr0077	1007	C	T	T	I	Histidine kinase
M68	spr0078	349	C	T	H	Y	30S Ribosomal protein S4
M50	spr0081	436	G	A	A	T	ABC transporter membrane-spanning permease - sugar transport
M32	spr0092	1211	C	T	A	V	CapD protein required for the biosynthesis of type 1 capsular polysaccharide
M41	spr0098	188	G	A	W	*	Hypothetical protein
M43	spr0099	400	C	T	L	F	ABC transporter membrane-spanning permease - amino acid transport
M66	spr0103	1316	C	T	S	F	Arginine succinate lyase
M67	spr0103	1316	C	T	S	F	Arginine succinate lyase
M69	spr0103	1316	C	T	S	F	Arginine succinate lyase
M6	spr0110	188	C	A	R	L	Hypothetical protein
M71	spr0111	337	G	A	D	N	Hypothetical protein
M72	spr0111	337	G	A	D	N	Hypothetical protein
M73	spr0111	337	G	A	D	N	Hypothetical protein
M46	spr0115	307	G	A	A	T	Hypothetical protein
M32	spr0123	335	C	T	P	L	Conserved hypothetical protein
M32	spr0124	1592	G	A	G	E	Glucose inhibited division protein A
M29	spr0131	230	C	T	T	I	Secreted metalloendopeptidase Gcp
M55	spr0131	310	G	A	A	T	Secreted metalloendopeptidase Gcp
M12	spr0137	353	C	T	T	I	ABC transporter ATP-binding membrane spanning permease - possible multidrug resistance
M45	spr0138	535	G	A	D	N	Hypothetical protein
M37	spr0158	145	G	A	Q	*	Hypothetical protein
M34	spr0162	887	C	T	R	Q	Riboflavin biosynthesis GTP-cyclohydrolase II
M51	spr0162	214	G	A	P	S	Riboflavin biosynthesis GTP-cyclohydrolase II
M56	spr0163	580	C	T	D	N	Riboflavin synthase alpha-chain
M32	spr0164	976	G	A	P	S	Riboflavin biosynthese a deaminase
M59	spr0170	260	G	A	T	I	Magnesium and cobalt transporter
M45	spr0174	425	C	T	T	M	Hypothetical protein
M52	spr0178	118	C	T	P	S	Dihydrofolate:folylpolyglutamate synthetase
M46	spr0181	130	C	T	P	S	orf47
M45	spr0183	2107	C	T	P	S	Ribonucleotide reductase class III anaerobic
M14	spr0189	211	G	A	G	R	50S Ribosomal protein L4
M15	spr0189	211	G	A	G	R	50S Ribosomal protein L4
M16	spr0189	211	G	A	G	R	50S Ribosomal protein L4
M17	spr0189	211	G	A	G	R	50S Ribosomal protein L4
M18	spr0189	200	A	G	Q	R	50S Ribosomal protein L4
M19	spr0189	211	G	A	G	R	50S Ribosomal protein L4
M20	spr0189	211	G	A	G	R	50S Ribosomal protein L4
M21	spr0189	211	G	A	G	R	50S Ribosomal protein L4

M24	spr0189	200	A	G	Q	R	50S Ribosomal protein L4
M25	spr0189	211	G	A	G	R	50S Ribosomal protein L4
M27	spr0189	211	G	A	G	R	50S Ribosomal protein L4
M28	spr0189	202	A	G	K	E	50S Ribosomal protein L4
M30	spr0189	211	G	A	G	R	50S Ribosomal protein L4
M33	spr0189	211	G	A	G	R	50S Ribosomal protein L4
M36	spr0189	211	G	A	G	R	50S Ribosomal protein L4
M42	spr0189	211	G	A	G	R	50S Ribosomal protein L4
M44	spr0189	211	G	A	G	R	50S Ribosomal protein L4
M45	spr0189	202	A	G	K	E	50S Ribosomal protein L4
M47	spr0189	211	G	A	G	R	50S Ribosomal protein L4
M48	spr0189	211	G	A	G	R	50S Ribosomal protein L4
M5	spr0189	211	G	A	G	R	50S Ribosomal protein L4
M59	spr0189	211	G	A	G	R	50S Ribosomal protein L4
M6	spr0189	211	G	A	G	R	50S Ribosomal protein L4
M64	spr0189	211	G	A	G	R	50S Ribosomal protein L4
M65	spr0189	211	G	A	G	R	50S Ribosomal protein L4
M7	spr0189	189	A	ACCATGG	na	na	50S Ribosomal protein L4
M71	spr0189	211	G	A	G	R	50S Ribosomal protein L4
M72	spr0189	211	G	A	G	R	50S Ribosomal protein L4
M73	spr0189	211	G	A	G	R	50S Ribosomal protein L4
M8	spr0189	211	G	A	G	R	50S Ribosomal protein L4
M34	spr0194	314	C	T	A	V	50S Ribosomal protein L22
M50	spr0205	130	G	A	V	M	50S Ribosomal protein L18
M47	spr0215	860	G	A	R	Q	RNA polymerase alpha subunit
M48	spr0215	860	G	A	R	Q	RNA polymerase alpha subunit
M68	spr0219	364	G	A	D	N	Phosphoglycerate mutase 2 paralog
M60	spr0226	278	C	T	G	E	Pyruvate formate-lyase 3
M63	spr0226	278	C	T	G	E	Pyruvate formate-lyase 3
M59	spr0228	139	G	A	A	T	Conserved hypothetical protein
M64	spr0228	353	G	A	G	E	Conserved hypothetical protein
M65	spr0228	353	G	A	G	E	Conserved hypothetical protein
M27	spr0231	1243	G	A	V	M	Phosphotransferase system sugar-specific EIIC component
M3	spr0233	382	G	A	D	N	Transaldolase C
M28	spr0234	76	G	A	D	N	Glycerol dehydrogenase NAD%2B dependent
M3	spr0234	652	G	A	A	T	Glycerol dehydrogenase NAD%2B dependent
M5	spr0234	1066	G	A	D	N	Glycerol dehydrogenase NAD%2B dependent
M50	spr0234	428	C	T	S	F	Glycerol dehydrogenase NAD%2B dependent
M31	spr0235	1009	G	A	V	I	Leucyl-tRNA synthetase
M55	spr0235	1003	G	A	G	S	Leucyl-tRNA synthetase
M50	spr0237	94	G	A	E	K	Conserved hypothetical protein
M56	spr0242	415	C	T	H	Y	Determinant for enhanced expression of pheromone
M47	spr0246	245	G	A	S	N	Conserved hypothetical protein
M48	spr0246	245	G	A	S	N	Conserved hypothetical protein
M29	spr0247	2503	G	A	P	S	Alkaline amylopullulanase
M29	spr0247	766	G	A	P	S	Alkaline amylopullulanase
M51	spr0247	2387	G	A	T	I	Alkaline amylopullulanase
M51	spr0250	1783	G	A	A	T	Elongation factor G
M56	spr0250	340	C	T	P	S	Elongation factor G
M33	spr0251	1255	G	A	D	N	DNA polymerase III alpha subunit
M46	spr0251	1919	C	T	T	I	DNA polymerase III alpha subunit
M5	spr0254	1087	G	A	A	T	Aminopeptidase
M58	spr0255	161	G	A	G	E	Conserved hypothetical protein
M61	spr0255	161	G	A	G	E	Conserved hypothetical protein
M62	spr0255	161	G	A	G	E	Conserved hypothetical protein
M59	spr0286	3010	C	T	P	S	Hyaluronate lyase precursor hyaluronidase hyase
M59	spr0291	293	C	T	A	V	Phosphotransferase system sugar-specific EII component
M20	spr0304	2119	G	A	A	T	Penicillin-binding protein 2X
M44	spr0309	367	C	T	A	T	Hypothetical protein
M29	spr0317	1013	G	A	G	E	The type 2 capsule locus of Streptococcus pneumoniae
M9	spr0319	346	C	A	H	N	The type 2 capsule locus of Streptococcus pneumoniae

M38	spr0322	109	G	GA	na	na	dTDP-glucose-4 6-dehydratase
M38	spr0323	205	G	A	E	K	dTDP-L-rhamnose synthase
M32	spr0328	3596	C	T	A	V	Conserved hypothetical protein
M55	spr0329	226	C	T	A	T	Penicillin-binding protein 1A
M21	spr0333	254	C	T	A	V	Conserved hypothetical protein
M27	spr0339	329	G	A	R	H	diphosphomevalonate decarboxylase
M29	spr0341	544	G	A	G	S	Isopentenyl diphosphate isomerase
M13	spr0356	502	G	A	E	K	Mannitol PTS EII
M37	spr0357	623	C	T	A	V	Conserved hypothetical protein
M24	spr0362	958	G	A	E	K	Trigger factor
M3	spr0362	580	G	A	G	S	Trigger factor
M59	spr0366	172	C	T	L	F	Hypothetical protein
M56	spr0371	212	G	A	G	E	Exfoliative toxin A
M55	spr0377	23	A	G	Q	R	3-oxoacyl-acyl-carrier-protein synthase III
M55	spr0381	389	C	T	A	V	3-ketoacyl-acyl carrier protein reductase
M5	spr0383	245	C	T	A	V	Biotin carboxyl carrier protein of acetyl-CoA carboxylase
M21	spr0386	314	G	A	R	H	Acetyl-coenzyme A carboxylase carboxyl transferase subunit beta
M46	spr0393	679	C	T	E	K	Glutamyl tRNA-Gln amidotransferase subunit B
M32	spr0397	292	G	A	E	K	Conserved hypothetical protein
M56	spr0398	79	C	T	R	C	50S Ribosomal protein L28
M5	spr0399	157	G	A	E	K	Alkaline shock protein
M29	spr0401	1630	G	A	E	K	Acetolactate synthase large subunit
M30	spr0405	139	C	T	P	S	Hypothetical protein
M27	spr0410	1883	G	A	G	E	Hypothetical protein
M21	spr0415	202	G	A	A	T	Pyruvate formate-lyase
M32	spr0422	925	G	A	D	N	Hypothetical protein
M44	spr0427	391	C	T	A	T	Trk transporter NAD%2B binding protein - K%2B transport
M20	spr0430	1588	A	G	T	A	ABC transporter ATP-binding protein - cobalt transport
M29	spr0430	1102	G	A	A	T	ABC transporter ATP-binding protein - cobalt transport
M32	spr0439	379	TA	T	na	na	Conserved hypothetical protein
M26	spr0441	985	C	T	P	S	Phosphoglycerate kinase
M38	spr0443	121	G	A	D	N	Transcriptional repressor of the glutamine synthetase gene
M59	spr0443	304	C	T	L	F	Transcriptional repressor of the glutamine synthetase gene
M50	spr0450	1499	C	T	G	E	EcoA type I restriction-modification enzyme R subunit
M55	spr0455	814	C	T	R	C	Class I heat-shock protein molecular chaperone
M57	spr0455	1193	C	T	T	I	Class I heat-shock protein molecular chaperone
M55	spr0469	590	C	T	G	E	Conserved hypothetical protein truncation
M6	spr0481	478	C	T	R	C	Initiation factor IF2
M36	spr0483	29	G	A	S	N	Conserved hypothetical protein
M44	spr0509	1811	G	A	G	D	Phenylalanyl-tRNA synthetase beta chain
M57	spr0522	409	C	T	P	S	Hypothetical protein
M60	spr0526	862	C	T	P	S	ABC transporter membrane-spanning permease - Pep export
M63	spr0526	862	C	T	P	S	ABC transporter membrane-spanning permease - Pep export
M55	spr0531	563	C	T	G	E	Hypothetical protein
M35	spr0532	54	T	G	K	N	ABC transporter membrane-spanning permease - glutamine transport
M55	spr0532	213	C	T	W	*	ABC transporter membrane-spanning permease - glutamine transport
M23	spr0540	844	G	A	E	K	Serine alanine adding enzyme
M51	spr0540	204	G	A	M	I	Serine alanine adding enzyme
M30	spr0544	590	G	A	R	H	Conserved hypothetical protein
M12	spr0551	139	G	A	G	S	Branched-chain amino acid transport system carrier protein
M4	spr0551	139	G	A	G	S	Branched-chain amino acid transport system carrier protein
M50	spr0558	845	C	T	T	I	Conserved hypothetical protein
M59	spr0558	785	G	A	W	*	Conserved hypothetical protein
M51	spr0561	1968	G	A	W	*	Cell wall-associated serine proteinase precursor PrtA
M13	spr0562	336	G	A	M	I	Phosphotransferase system sugar-specific EII component
M28	spr0565	3056	G	A	G	D	beta-galactosidase precursor
M29	spr0565	1219	C	T	R	C	beta-galactosidase precursor
M30	spr0579	1607	G	A	G	E	Histidine kinase
M59	spr0580	358	G	A	A	T	Conserved hypothetical protein
M12	spr0581	2609	G	T	G	V	Zinc metalloprotease
M14	spr0581	3664	G	A	V	M	Zinc metalloprotease

M29	spr0581	4412	G	A	G	E	Zinc metalloprotease
M30	spr0581	823	G	A	E	K	Zinc metalloprotease
M43	spr0590	598	C	T	R	C	Hypothetical protein
M14	spr0602	146	G	A	R	Q	ABC transporter ATP-binding protein - sodium transport
M52	spr0603	1271	C	T	A	V	UDP-N-acetylmuramoyl-L-alanine--D-glutamate ligase
M46	spr0605	328	G	A	A	T	Cell division protein DivIB
M53	spr0631	343	G	A	G	R	Conserved hypothetical protein
M54	spr0631	343	G	A	G	R	Conserved hypothetical protein
M6	spr0636	737	A	C	N	T	Hydroxyethylthiazole kinase
M51	spr0637	119	G	A	R	Q	Thiamine phosphate pyrophosphorylase
M57	spr0640	151	C	T	P	S	Conserved hypothetical protein
M38	spr0646	151	G	A	A	T	Phospho-beta-gluco or galactosidase truncation
M51	spr0652	449	G	A	T	I	Conserved hypothetical protein
M36	spr0653	164	G	A	G	D	Conserved hypothetical protein
M52	spr0654	68	C	T	S	F	dCMP deaminase
M51	spr0660	71	G	A	G	E	ABC transporter membrane-spanning permease - branched chain amino acid transport
M16	spr0661	503	C	T	T	I	ABC transporter membrane-spanning permease - branched chain amino acid transport
M35	spr0665	216	G	A	M	I	Peptide chain release factor 2
M59	spr0668	948	G	A	M	I	PTS glucose-specific enzyme IIABC component
M51	spr0671	866	A	G	Y	C	S-adenosylmethionine synthetase
M27	spr0673	688	G	A	D	N	DNA polymerase III delta subunit
M15	spr0676	39	G	A	M	I	Conserved hypothetical protein
M35	spr0678	1522	G	A	D	N	ABC transporter ATP-binding protein - unknown substrate
M52	spr0681	67	C	T	D	N	Hypothetical protein
M43	spr0693	935	G	A	G	D	Conserved hypothetical protein
M37	spr0694	623	C	T	A	V	ABC transporter ATP-binding protein - unknown substrate
M31	spr0708	1087	C	T	L	F	Sensor protein CiaH histide kinase
M59	spr0712	445	G	A	E	K	Rod shape determining protein
M23	spr0713	148	G	A	A	T	4-methyl-5 b-hydroxyethyl -thiazole monophosphate biosynthesis protein
M59	spr0756	283	G	A	V	I	Topoisomerase IV subunit B
M44	spr0757	1549	C	T	R	C	Topoisomerase IV subunit A
M6	spr0757	479	C	T	A	V	Topoisomerase IV subunit A
M44	spr0762	427	C	T	H	Y	Pyrrolidone-carboxylate peptidase 5-oxoprolyl-peptidase
M59	spr0762	577	G	A	V	I	Pyrrolidone-carboxylate peptidase 5-oxoprolyl-peptidase
M57	spr0767	407	C	T	R	H	Transposase orf1
M27	spr0773	1120	C	T	P	S	Conserved hypothetical protein
M57	spr0784	931	C	T	P	S	Thiamin biosynthesis protein
M60	spr0785	35	C	T	S	F	Hypothetical protein
M63	spr0785	35	C	T	S	F	Hypothetical protein
M59	spr0790	1191	G	A	M	I	Type I restriction modification enzyme methylase subunit
M55	spr0792	3059	A	G	K	R	Type 1 restriction modification system endonuclease R
M13	spr0796	811	C	T	H	Y	6-phosphofructokinase I
M55	spr0804	379	C	T	D	N	Conserved hypothetical protein
M27	spr0808	1081	G	A	D	N	Toxin expression - transcriptional accessory protein
M37	spr0813	1585	G	A	A	T	ABC transporter membrane-spanning permease - unknown substrate
M38	spr0819	220	C	T	P	S	Spermidine synthase
M42	spr0821	147	T	A	Y	*	Carboxynorspermidine decarboxylase
M57	spr0821	466	C	T	P	S	Carboxynorspermidine decarboxylase
M5	spr0827	17	G	A	P	L	Hypothetical protein
M27	spr0832	622	G	A	A	T	Gamma-glutamyl kinase
M23	spr0856	577	G	A	D	N	Competence protein
M50	spr0856	352	G	A	A	T	Competence protein
M46	spr0857	1727	G	A	G	E	Competence protein
M55	spr0865	587	A	T	D	V	Dihydroorotate dehydrogenase electron transfer subunit
M51	spr0867	739	G	A	A	T	Endo-beta-N-acetylglucosaminidase
M53	spr0875	803	G	A	G	E	Major facilitator superfamily multi-drug resistance efflux pump
M54	spr0875	803	G	A	G	E	Major facilitator superfamily multi-drug resistance efflux pump
M50	spr0878	2293	G	A	E	K	Exoribonuclease R
M56	spr0882	1661	G	A	G	D	Group B oligopeptidase
M45	spr0890	54	C	T	M	I	Hypothetical protein
M28	spr0906	10	G	A	E	K	Lipoprotein

M52	spr0906	794	C	T	S	L	Lipoprotein
M23	spr0946	847	G	A	A	T	Conserved hypothetical protein
M37	spr0950	272	C	T	S	F	Hypothetical protein
M46	spr0958	48	G	A	M	I	Tn5252 relaxase truncation
M16	spr0965	1057	C	T	P	S	Hypothetical protein
M50	spr0965	1057	C	T	P	S	Hypothetical protein
M38	spr0973	461	G	A	R	H	Cell division protein FtsW
M47	spr0974	346	G	A	V	I	Phosphoenolpyruvate carboxylase
M48	spr0974	346	G	A	V	I	Phosphoenolpyruvate carboxylase
M50	spr0974	484	C	T	R	C	Phosphoenolpyruvate carboxylase
M64	spr0982	869	C	T	A	V	Conserved Hypothetical protein
M65	spr0982	869	C	T	A	V	Conserved Hypothetical protein
M32	spr0993	172	C	T	P	S	Degenerate transposase orf1
M38	spr0993	88	G	A	G	S	Degenerate transposase orf1
M20	spr0995	1760	C	T	A	V	ATP-dependent DNA helicase
M38	spr0995	2278	G	A	E	K	ATP-dependent DNA helicase
M66	spr1005	265	G	A	D	N	Conserved hypothetical protein
M67	spr1005	265	G	A	D	N	Conserved hypothetical protein
M69	spr1005	265	G	A	D	N	Conserved hypothetical protein
M64	spr1009	212	G	A	T	I	Transposase orf1
M65	spr1009	212	G	A	T	I	Transposase orf1
M10	spr1019	655	C	T	L	F	Conserved hypothetical protein
M7	spr1021	919	G	A	A	T	ABC transporter ATP-binding protein - unknown substrate
M46	spr1039	1798	G	A	V	I	Second subunit of major exonuclease
M52	spr1041	766	C	T	L	F	Hypothetical protein
M44	spr1042	4651	G	A	E	K	Immunoglobulin A1 protease
M51	spr1042	3856	G	A	A	T	Immunoglobulin A1 protease
M18	spr1046	94	C	A	P	T	Integrase recombinase
M47	spr1047	653	G	T	S	*	Lipoate protein ligase A
M48	spr1047	653	G	T	S	*	Lipoate protein ligase A
M45	spr1048	232	C	T	E	K	Dihydrolipoamide dehydrogenase
M22	spr1056	188	G	A	S	F	Hypothetical protein
M25	spr1059	140	C	T	G	E	Hypothetical protein
M57	spr1060	827	C	T	G	E	Histidine Motif-Containing protein
M55	spr1062	853	C	T	G	S	PTS enzyme I
M37	spr1065	632	C	T	A	V	Ribonucleoside-diphosphate reductase major subunit
M46	spr1065	1687	G	A	D	N	Ribonucleoside-diphosphate reductase major subunit
M52	spr1090	149	G	A	T	I	Uridine kinase
M17	spr1092	35	C	T	G	E	tRNA pseudouridine 5S synthase
M58	spr1092	553	C	T	G	S	tRNA pseudouridine 5S synthase
M61	spr1092	553	C	T	G	S	tRNA pseudouridine 5S synthase
M62	spr1092	553	C	T	G	S	tRNA pseudouridine 5S synthase
M52	spr1093	949	G	A	L	F	Conserved hypothetical protein
M55	spr1093	2558	G	A	A	V	Conserved hypothetical protein
M64	spr1093	1906	G	T	Q	K	Conserved hypothetical protein
M65	spr1093	1906	G	T	Q	K	Conserved hypothetical protein
M29	spr1100	100	G	A	E	K	L-lactate dehydrogenase
M45	spr1108	244	G	A	P	S	Similar to A G-specific adenine glycosylase
M15	spr1120	1953	G	A	M	I	ABC transporter membrane spanning permease - glutamine transport
M24	spr1120	1856	C	A	A	D	ABC transporter membrane spanning permease - glutamine transport
M18	spr1121	535	G	A	V	I	ABC transporter ATP-binding protein - glutamine transport
M19	spr1121	697	C	G	H	D	ABC transporter ATP-binding protein - glutamine transport
M25	spr1121	137	C	G	T	S	ABC transporter ATP-binding protein - glutamine transport
M5	spr1121	272	A	G	N	S	ABC transporter ATP-binding protein - glutamine transport
M43	spr1122	34	G	A	A	T	Glucose-6-phosphate 1-dehydrogenase
M45	spr1126	530	G	A	T	I	chromosome condensation and segregation SMC protein
M51	spr1129	1139	G	A	A	V	Hypothetical protein
M43	spr1146	142	C	T	D	N	Homologous to LicB which regulates expression of LPS epitopes in H. influenzae
M28	spr1148	584	G	A	P	L	Conserved hypothetical protein
M60	spr1154	383	C	T	R	H	Carbamoylphosphate synthase glutamine-hydrolysing light subunit
M63	spr1154	383	C	T	R	H	Carbamoylphosphate synthase glutamine-hydrolysing light subunit

M17	spr1161	662	C	T	A	V	ABC transporter ATP-binding protein - unknown substrate
M39	spr1170	721	C	T	P	S	Conserved hypothetical protein
M46	spr1171	7	G	A	P	S	50S Ribosomal protein L19
M16	spr1181	1121	C	T	A	V	NADP-specific glutamate dehydrogenase
M52	spr1183	336	C	T	M	I	ABC transporter ATP-bindng protein - possibly multidrug efflux truncation
M55	spr1183	14	G	A	P	L	ABC transporter ATP-bindng protein - possibly multidrug efflux truncation
M55	spr1184	181	G	A	A	T	Hypothetical protein
M35	spr1192	235	C	T	G	S	ABC transporter membrane-spanning permease - oligopeptide transport
M55	spr1205	1426	G	A	Q	*	Conserved hypothetical protein
M50	spr1211	355	C	T	V	I	50S Ribosomal protein L12
M58	spr1215	563	G	A	S	F	ABC transporter ATP-binding membrane-spanning protein - unknown substrate
M61	spr1215	563	G	A	S	F	ABC transporter ATP-binding membrane-spanning protein - unknown substrate
M62	spr1215	563	G	A	S	F	ABC transporter ATP-binding membrane-spanning protein - unknown substrate
M66	spr1218	614	C	T	G	E	Homoserine kinase
M67	spr1218	614	C	T	G	E	Homoserine kinase
M69	spr1218	614	C	T	G	E	Homoserine kinase
M17	spr1222	1382	C	T	G	E	Hypothetical protein
M25	spr1231	445	G	A	P	S	Prephenate dehydrogenase
M44	spr1239	1232	G	A	G	D	Alpha-amylase precursor
M64	spr1239	124	C	A	P	T	Alpha-amylase precursor
M65	spr1239	124	C	A	P	T	Alpha-amylase precursor
M52	spr1250	781	G	A	G	S	Conserved hypothetical protein
M58	spr1259	82	C	T	E	K	Conserved hypothetical protein
M61	spr1259	82	C	T	E	K	Conserved hypothetical protein
M62	spr1259	82	C	T	E	K	Conserved hypothetical protein
M23	spr1266	464	G	A	S	F	Coproporphyrinogen III oxidase
M56	spr1267	41	C	T	G	D	Conserved hypothetical protein
M43	spr1269	599	C	T	R	Q	Prolipoprotein diacylglycerol transferase
M23	spr1273	556	C	T	L	F	S-adenosylmethionine:tRNA ribosyltransferase-isomerase
M47	spr1273	43	C	T	P	S	S-adenosylmethionine:tRNA ribosyltransferase-isomerase
M48	spr1273	43	C	T	P	S	S-adenosylmethionine:tRNA ribosyltransferase-isomerase
M52	spr1277	115	G	A	R	C	Conserved hypothetical protein
M20	spr1278	416	C	T	G	D	Hypothetical protein
M32	spr1289	689	C	T	S	L	ABC transporter ATP-binding membrane-spanning protein - unknown substrate
M31	spr1290	410	C	T	A	V	ABC transporter ATP-binding membrane-spanning protein - unknown substrate
M64	spr1290	299	G	T	R	I	ABC transporter ATP-binding membrane-spanning protein - unknown substrate
M65	spr1290	299	G	T	R	I	ABC transporter ATP-binding membrane-spanning protein - unknown substrate
M59	spr1301	598	G	A	A	T	Conserved hypothetical protein
M60	spr1306	478	C	T	V	I	Conserved hypothetical protein
M63	spr1306	478	C	T	V	I	Conserved hypothetical protein
M50	spr1318	226	C	T	E	K	Hypothetical protein
M3	spr1326	398	C	T	A	V	Hypothetical protein
M30	spr1335	932	C	T	A	V	Oxidoreductase
M50	spr1346	64	C	T	A	T	Hypothetical protein
M26	spr1355	343	C	T	A	T	ABC transporter membrane-spanning permease - glutamine transport
M35	spr1356	259	C	T	A	T	Conserved hypothetical protein
M55	spr1357	1094	G	A	A	V	Conserved hypothetical protein
M27	spr1362	1469	G	A	A	V	Proton-translocating ATPase F1 sector alpha-subunit
M37	spr1364	448	G	A	H	Y	Proton-translocating ATPase F0 sector subunit b
M41	spr1364	292	C	T	D	N	Proton-translocating ATPase F0 sector subunit b
M11	spr1367	304	G	A	Q	*	Transposase
M51	spr1367	121	G	A	P	S	Transposase
M71	spr1374	281	T	G	K	T	Hypothetical protein
M72	spr1374	281	T	G	K	T	Hypothetical protein
M73	spr1374	281	T	G	K	T	Hypothetical protein
M58	spr1375	2476	C	T	E	K	SWF SNF family ATP-dependent RNA helicase
M61	spr1375	2476	C	T	E	K	SWF SNF family ATP-dependent RNA helicase
M62	spr1375	2476	C	T	E	K	SWF SNF family ATP-dependent RNA helicase
M25	spr1379	428	GC	G	na	na	ABC transporter truncation
M30	spr1380	209	C	T	S	N	ABC transporter truncation
M37	spr1386	121	C	T	A	T	Hypothetical protein

M24	spr1404	91	C	T	R	C	Conserved hypothetical protein
M59	spr1404	214	G	A	G	R	Conserved hypothetical protein
M30	spr1410	479	C	T	R	H	P-type ATPase - calcium transporter
M32	spr1412	1037	C	T	G	E	ABC transporter ATP-binding protein - unknown substrate
M7	spr1412	1591	T	C	T	A	ABC transporter ATP-binding protein - unknown substrate
M32	spr1416	286	C	T	V	M	Hypothetical protein
M58	spr1428	31	C	T	A	T	Hypothetical protein
M61	spr1428	31	C	T	A	T	Hypothetical protein
M62	spr1428	31	C	T	A	T	Hypothetical protein
M47	spr1431	504	C	T	W	*	1 4-beta-N-acetylmuramidase
M48	spr1431	504	C	T	W	*	1 4-beta-N-acetylmuramidase
M51	spr1440	926	G	A	T	I	Conserved hypothetical protein
M66	spr1443	572	G	A	G	E	Conserved hypothetical protein
M67	spr1443	572	G	A	G	E	Conserved hypothetical protein
M69	spr1443	572	G	A	G	E	Conserved hypothetical protein
M17	spr1451	730	C	T	E	K	tRNA pseudouridine synthase A
M51	spr1475	378	G	A	W	*	Hypothetical protein
M46	spr1482	195	G	A	W	*	Hypothetical protein
M12	spr1491	1394	G	A	S	F	Endopeptidase O
M4	spr1491	1394	G	A	S	F	Endopeptidase O
M27	spr1496	1894	C	T	G	R	Hypothetical protein
M16	spr1502	901	G	A	P	S	Isoleucyl-tRNA synthetase
M55	spr1502	383	G	C	A	G	Isoleucyl-tRNA synthetase
M55	spr1505	655	G	A	R	C	Cell-division initiation protein septum placement
M51	spr1507	142	G	A	L	F	Conserved hypothetical protein
M55	spr1507	251	G	A	A	V	Conserved hypothetical protein
M66	spr1509	95	G	A	A	V	Conserved hypothetical protein
M67	spr1509	95	G	A	A	V	Conserved hypothetical protein
M69	spr1509	95	G	A	A	V	Conserved hypothetical protein
M64	spr1512	489	C	T	M	I	Hypothetical protein
M65	spr1512	489	C	T	M	I	Hypothetical protein
M8	spr1514	602	C	T	G	E	UDP-N-acetylmuramoylalanine-D-glutamyl-lysine-D-alanyl-D-alanine ligase
M68	spr1516	506	C	T	R	H	Recombination protein RecR
M29	spr1517	365	G	A	A	V	Penicillin-binding protein 2B
M52	spr1517	1049	G	A	A	V	Penicillin-binding protein 2B
M58	spr1522	202	C	T	V	I	Hypothetical protein
M61	spr1522	202	C	T	V	I	Hypothetical protein
M62	spr1522	202	C	T	V	I	Hypothetical protein
M16	spr1525	148	G	A	P	S	ABC transporter membrane-spanning permease - sugar transport
M45	spr1525	160	G	A	P	S	ABC transporter membrane-spanning permease - sugar transport
M36	spr1530	748	G	A	R	C	Conserved hypothetical protein
M37	spr1530	539	C	T	C	Y	Conserved hypothetical protein
M43	spr1534	673	G	A	L	F	ABC transporter substrate-binding protein - sugar transport
M13	spr1536	865	G	A	L	F	Sialidase A precursor neuraminidase A
M13	spr1543	235	G	A	R	C	phospho-2-dehydro-3-deoxyheptonate aldolase DAHP synthatase isozyme - possibly phe sensitive
M38	spr1544	773	G	A	T	I	Preprotein translocase secA subunit
M32	spr1545	28	TC	T	na	na	Hypothetical protein
M60	spr1545	28	T	TC	na	na	Hypothetical protein
M63	spr1545	28	T	TC	na	na	Hypothetical protein
M12	spr1546	22	C	T	G	R	ABC transporter ATP-binding protein - unknown substrate
M4	spr1546	22	C	T	G	R	ABC transporter ATP-binding protein - unknown substrate
M59	spr1546	367	C	T	E	K	ABC transporter ATP-binding protein - unknown substrate
M59	spr1566	1338	C	T	M	I	Phosphotransferase system enzyme II
M44	spr1570	1033	G	A	P	S	3-hydroxy-3-methylglutaryl-coenzyme a reductase
M1	spr1575	398	C	T	G	E	Hypothetical protein
M51	spr1579	716	G	A	A	V	rRNA methylase
M21	spr1585	211	C	T	D	N	Conserved hypothetical protein
M50	spr1587	394	G	A	Q	*	Conserved hypothetical protein
M12	spr1589	187	C	T	G	S	Conserved hypothetical protein
M4	spr1589	187	C	T	G	S	Conserved hypothetical protein
M47	spr1592	601	C	T	D	N	Conserved hypothetical protein

M48	spr1592	601	C	T	D	N	Conserved hypothetical protein
M46	spr1599	586	G	A	V	I	Conserved hypothetical protein
M59	spr1603	211	C	T	R	C	Conserved hypothetical protein
M55	spr1608	766	C	T	A	T	Ribosomal protein methyltransferase
M46	spr1617	845	G	A	A	V	Sucrose-6-phosphate hydrolase
M50	spr1621	366	C	T	M	I	Sucrose operon repressor SCR operon regulatory protein
M20	spr1622	1411	C	T	D	N	Conserved hypothetical protein
M32	spr1625	142	C	T	L	F	Conserved hypothetical protein
M17	spr1630	305	G	A	P	L	Hypothetical protein
M41	spr1630	667	C	T	E	K	Hypothetical protein
M46	spr1652	764	C	T	G	D	Hypothetical protein
M47	spr1654	1046	G	A	A	V	Conserved hypothetical protein
M48	spr1654	1046	G	A	A	V	Conserved hypothetical protein
M35	spr1662	281	C	G	A	G	Xanthine phosphoribosyltransferase
M56	spr1668	917	G	A	A	V	Galactokinase
M43	spr1682	778	C	T	D	N	Glutamyl aminopeptidase
M55	spr1684	956	G	A	G	E	ABC transporter membrane-spanning permease - ferric iron transport
M6	spr1698	191	G	A	S	L	Dextran glucosidase
M64	spr1703	647	C	T	G	D	ABC transporter ATP-binding protein - oligopeptide transport
M65	spr1703	647	C	T	G	D	ABC transporter ATP-binding protein - oligopeptide transport
M55	spr1704	796	G	A	L	F	ABC transporter ATP-binding protein - oligopeptide transport
M47	spr1708	997	G	A	R	C	Hypothetical protein
M48	spr1708	997	G	A	R	C	Hypothetical protein
M44	spr1733	136	C	T	V	I	Hypothetical protein
M41	spr1734	673	C	T	D	N	ABC transporter ATP-binding protein - unknown substrate truncation
M55	spr1734	64	G	A	R	W	ABC transporter ATP-binding protein - unknown substrate truncation
M46	spr1739	194	G	A	T	I	Pneumolysin sulfhydryl-activated toxin that lyses cholesterol containing membranes
M29	spr1748	45	C	T	W	*	Hypothetical protein
M8	spr1759	586	C	T	E	K	Regulator of lytRABC operon
M60	spr1767	898	G	A	D	N	CylM protein cytolytic toxin system
M63	spr1767	898	G	A	D	N	CylM protein cytolytic toxin system
M44	spr1776	3520	G	A	P	S	DNA-dependent RNA polymerase
M50	spr1776	2440	G	A	R	C	DNA-dependent RNA polymerase
M51	spr1776	3064	G	A	R	C	DNA-dependent RNA polymerase
M64	spr1776	3532	C	T	G	S	DNA-dependent RNA polymerase
M65	spr1776	3532	C	T	G	S	DNA-dependent RNA polymerase
M18	spr1777	1297	G	A	L	F	DNA-dependent RNA polymerase subunit beta
M34	spr1777	895	C	T	V	I	DNA-dependent RNA polymerase subunit beta
M55	spr1783	214	G	A	H	Y	lipopolysaccharide core biosynthesis protein
M29	spr1784	272	C	T	G	E	Conserved hypothetical protein
M51	spr1792	1180	G	A	R	C	Diaminopimelate decarboxylase
M56	spr1795	577	C	T	G	R	Conserved hypothetical protein
M51	spr1799	43	G	A	R	C	Dimethyladenosine transferase
M71	spr1805	253	A	G	S	P	Conserved hypothetical protein
M72	spr1805	253	A	G	S	P	Conserved hypothetical protein
M73	spr1805	253	A	G	S	P	Conserved hypothetical protein
M17	spr1811	400	G	A	Q	*	Conserved hypothetical protein
M50	spr1812	238	G	A	V	M	L-asparaginase L-asparagine amidohydrolase
M44	spr1813	457	G	A	R	C	Catabolite control protein A
M51	spr1823	1138	C	T	V	I	Penicillin-binding protein 2a
M8	spr1823	1576	C	A	G	C	Penicillin-binding protein 2a
M57	spr1824	677	G	A	R	Q	Ribosomal large subunit pseudouridine synthase D
M10	spr1830	285	G	A	M	I	Conserved hypothetical protein
M51	spr1833	821	G	A	G	E	Beta-glucosidase
M35	spr1837	19	TTTCCTCCA	T	na	na	Alcohol-acetaldehyde dehydrogenase
M56	spr1837	398	T	G	N	T	Alcohol-acetaldehyde dehydrogenase
M47	spr1842	844	G	A	R	C	Conserved hypothetical protein
M48	spr1842	844	G	A	R	C	Conserved hypothetical protein
M28	spr1843	1163	C	T	G	E	Conserved hypothetical protein
M59	spr1844	340	G	A	P	S	L-ribulose 5-phosphate 4-epimerase
M16	spr1858	50	G	A	T	I	Hypothetical protein

M56	spr1863	397	C	T	A	T	Competence protein
M32	spr1864	22	C	T	E	K	Competence protein
M34	spr1866	326	C	A	G	V	Putative alcohol dehydrogenase
M37	spr1866	686	AT	A	na	na	Putative alcohol dehydrogenase
M46	spr1869	737	G	A	P	L	tRNA-guanine transglycosylase guanine insertion enzyme
M32	spr1875	68	G	A	A	V	Conserved hypothetical protein
M47	spr1881	349	C	T	E	K	Glutamyl-tRNA synthetase glutamate--tRNA ligase
M48	spr1881	349	C	T	E	K	Glutamyl-tRNA synthetase glutamate--tRNA ligase
M50	spr1881	723	A	T	F	L	Glutamyl-tRNA synthetase glutamate--tRNA ligase
M34	spr1889	88	C	T	E	K	Arginine repressor
M20	spr1890	1135	G	A	V	I	Arginyl-tRNA synthetase arginine--tRNA ligase ARGRS
M7	spr1896	704	C	T	S	L	ABC transporter membrane-spanning permease - phosphate transport
M37	spr1899	3	G	A	M	I	Negative regulator of pho regulon for phosphate transport
M8	spr1908	416	G	A	S	L	Conserved hypothetical protein
M50	spr1927	469	G	A	R	C	Hypothetical protein
M42	spr1929	166	T	C	I	V	Conserved hypothetical protein
M51	spr1932	743	G	A	S	F	Conserved hypothetical protein
M32	spr1939	182	G	A	S	L	Hypothetical protein
M50	spr1941	671	G	A	S	N	Hypothetical protein
M45	spr1946	487	C	T	P	S	Degenerate transposase orf1
M20	spr1949	795	C	T	M	I	Hypothetical protein
M3	spr1958	944	G	A	G	E	Carbamate kinase
M56	spr1959	302	G	A	G	D	Conserved hypothetical integral membrane protein
M64	spr1964	64	C	T	E	K	L-fucose isomerase
M65	spr1964	64	C	T	E	K	L-fucose isomerase
M52	spr1965	2005	G	A	L	F	Hypothetical protein
M56	spr1967	512	G	A	T	I	Phosphotransferase system sugar-specific EII component
M50	spr1973	769	G	A	P	S	Fucose kinase
M16	spr1974	295	G	A	D	N	Regulator of fucose operon
M12	spr1979	1160	G	GCCAACCAAC	na	na	D-alanine transfer from undecaprenol-phosphate to the poly glycerophosphate chain
M55	spr1992	680	C	T	R	Q	Hypothetical protein
M37	spr2012	188	C	T	W	*	Involved in transformation competence for DNA uptake
M37	spr2028	199	C	T	E	K	Conserved hypothetical protein
M15	spr2029	370	C	T	D	N	Conserved hypothetical protein
M1	sprrRNA	2060	A	T	na	na	23S ribosomal RNA
M10	sprrRNA	2060	A	T	na	na	23S ribosomal RNA
M11	sprrRNA	2060	A	T	na	na	23S ribosomal RNA
M12	sprrRNA	2061	A	C	na	na	23S ribosomal RNA
M13	sprrRNA	2060	A	T	na	na	23S ribosomal RNA
M2	sprrRNA	2060	A	T	na	na	23S ribosomal RNA
M22	sprrRNA	2613	C	T	na	na	23S ribosomal RNA
M23	sprrRNA	2613	C	T	na	na	23S ribosomal RNA
M26	sprrRNA	2060	A	T	na	na	23S ribosomal RNA
M3	sprrRNA	2060	A	T	na	na	23S ribosomal RNA
M4	sprrRNA	2061	A	C	na	na	23S ribosomal RNA
M9	sprrRNA	2061	A	G	na	na	23S ribosomal RNA
M29	sprrRNA	2613	C	T	na	na	23S ribosomal RNA
M32	sprrRNA	2613	C	T	na	na	23S ribosomal RNA
M35	sprrRNA	2613	C	T	na	na	23S ribosomal RNA
M38	sprrRNA	2613	C	T	na	na	23S ribosomal RNA
M41	sprrRNA	2613	C	T	na	na	23S ribosomal RNA
M43	sprrRNA	2613	C	T	na	na	23S ribosomal RNA
M46	sprrRNA	2613	C	T	na	na	23S ribosomal RNA
M49	sprrRNA	2061	A	G	na	na	23S ribosomal RNA
M50	sprrRNA	2613	C	T	na	na	23S ribosomal RNA
M51	sprrRNA	2613	C	T	na	na	23S ribosomal RNA
M52	sprrRNA	2613	C	T	na	na	23S ribosomal RNA
M53	sprrRNA	2613	C	T	na	na	23S ribosomal RNA
M54	sprrRNA	2613	C	T	na	na	23S ribosomal RNA
M55	sprrRNA	2613	C	T	na	na	23S ribosomal RNA
M57	sprrRNA	2613	C	T	na	na	23S ribosomal RNA

M58	sprrRNA	2613	C	T	na	na	23S ribosomal RNA
M60	sprrRNA	2613	C	T	na	na	23S ribosomal RNA
M61	sprrRNA	2613	C	T	na	na	23S ribosomal RNA
M62	sprrRNA	2613	C	T	na	na	23S ribosomal RNA
M63	sprrRNA	2613	C	T	na	na	23S ribosomal RNA
M66	sprrRNA	2613	C	T	na	na	23S ribosomal RNA
M67	sprrRNA	2613	C	T	na	na	23S ribosomal RNA
M68	sprrRNA	2613	C	T	na	na	23S ribosomal RNA
M69	sprrRNA	2613	C	T	na	na	23S ribosomal RNA

Table S3. Mutations detected in the intergenic regions AZM-resistant mutants. Lines are sorted by intergenic region identifier to ease visualisation of recurrent regions.

Mutant	Intergenic region identifier	Chr position	Wild-type	Mutant	Gene up	Strand gene up	Gene down	Strand gene down	Function gene upstream	Function gene downstream
M44	intergenic_spr0057_spr0058	64580	G	A	spr0057	-	spr0058	-	Beta-N-acetyl-hexosaminidase precursor	Conserved hypothetical protein
M16	intergenic_spr0058_spr0059	65827	C	T	spr0058	-	spr0059	+	Conserved hypothetical protein	Beta-galactosidase 3
M59	intergenic_spr0067_spr0068	75073	G	A	spr0067	+	spr0068	+	Conserved hypothetical protein	Conserved hypothetical protein
M7	intergenic_spr0075_spr0076	83700	G	A	spr0075	+	spr0076	+	Conserved hypothetical protein	Response regulator
M3	intergenic_spr0133_spr0134	141686	G	A	spr0133	-	spr0134	-	Degenerate transposase orf2	Degenerative transposase
M55	intergenic_spr0137_spr0138	146396	G	A	spr0137	+	spr0138	+	ABC transporter ATP-binding membrane spanning perm	Hypothetical protein
M46	intergenic_spr0145_spr0146	154372	C	T	spr0145	+	spr0146	+	Hypothetical protein	ABC transporter substrate-binding protein - amino acid transport
M52	intergenic_spr0159_spr0160	167883	G	A	spr0159	-	spr0160	+	Conserved hypothetical protein	DNA mismatch repair protein
M55	intergenic_spr0186_spr0187	195498	G	A	spr0186	+	spr0187	+	Hypothetical protein	30S Ribosomal protein S10
M56	intergenic_spr0239_spr0240	233742	C	T	spr0239	+	spr0240	+	Hypothetical protein	Undecaprenyl diphosphate synthase
M14	intergenic_spr0263_spr0264	265581	G	A	spr0263	-	spr0264	+	Conserved hypothetical protein	Conserved hypothetical protein
M43	intergenic_spr0263_spr0264	265558	G	A	spr0263	-	spr0264	+	Conserved hypothetical protein	Conserved hypothetical protein
M20	intergenic_spr0265_spr0266	267997	G	A	spr0265	+	spr0266	+	Conserved hypothetical protein	Dihydropteroate synthase
M27	intergenic_spr0336_spr0337	341947	G	A	spr0336	+	spr0337	+	Response regulator	Choline-binding protein F
M15	intergenic_spr0414_spr0415	411943	G	A	spr0414	-	spr0415	+	DNA-damage-inducible protein P	Pyruvate formate-lyase
M30	intergenic_spr0416_spr0417	415305	C	T	spr0416	-	spr0417	-	Hypothetical protein	Conserved hypothetical protein
M6	intergenic_spr0442_spr0443	448043	G	A	spr0442	+	spr0443	+	Hypothetical protein	Transcriptional repressor of the glutamine synthetase gene
M5	intergenic_spr0444_spr0445	450588	C	T	spr0444	+	spr0445	+	Glutamine synthetase type 1	type I restriction enzyme
M14	intergenic_spr0444_spr0445	450588	C	T	spr0444	+	spr0445	+	Glutamine synthetase type 1	type I restriction enzyme
M16	intergenic_spr0444_spr0445	450588	C	T	spr0444	+	spr0445	+	Glutamine synthetase type 1	type I restriction enzyme
M25	intergenic_spr0444_spr0445	450588	C	T	spr0444	+	spr0445	+	Glutamine synthetase type 1	type I restriction enzyme
M57	intergenic_spr0444_spr0445	450588	C	T	spr0444	+	spr0445	+	Glutamine synthetase type 1	type I restriction enzyme
M62	intergenic_spr0444_spr0445	450588	C	T	spr0444	+	spr0445	+	Glutamine synthetase type 1	type I restriction enzyme
M31	intergenic_spr0504_spr0505	501728	C	T	spr0504	+	spr0505	+	Transcriptional antiterminator BglG family	Phosphotransferase system sugar-specific EII component
M68	intergenic_spr0506_spr0507	505373	C	T	spr0506	+	spr0507	+	6-phospho-beta-glucosidase	Phenylalanyl-tRNA synthetase alpha chain
M44	intergenic_spr0523_spr0524	523447	G	A	spr0523	+	spr0524	+	Transposase uncharacterized truncation	ABC transporter membrane-spanning permease - Pep export
M16	intergenic_spr0537_spr0538	537693	G	C	spr0537	+	spr0538	+	Single-stranded DNA-specific exonuclease 5%27-3%27	Conserved hypothetical protein
M52	intergenic_spr0548_spr0549	550163	G	A	spr0548	+	spr0549	+	Conserved hypothetical protein	Hypothetical protein
M59	intergenic_spr0554_spr0555	554722	G	A	spr0554	+	spr0555	+	Conserved hypothetical protein	50S Ribosomal protein L11
M50	intergenic_spr0556_spr0557	557528	C	T	spr0556	+	spr0557	+	50S Ribosomal protein L1	ABC transporter ATP-binding protein - role in polysaccharide efflux
M30	intergenic_spr0574_spr0575	585593	G	A	spr0574	+	spr0575	+	Conserved hypothetical protein	Cytochrome c-type biogenesis protein
M23	intergenic_spr0626_spr0627	634365	G	A	spr0626	+	spr0627	+	Lysyl-tRNA synthetase lysine--tRNA ligase LYSRS	Lactate oxidase
M26	intergenic_spr0627_spr0628	636180	A	G	spr0627	+	spr0628	+	Lactate oxidase	Conserved hypothetical protein
M60	intergenic_spr0627_spr0628	636173	C	T	spr0627	+	spr0628	+	Lactate oxidase	Conserved hypothetical protein
M63	intergenic_spr0627_spr0628	636173	C	T	spr0627	+	spr0628	+	Lactate oxidase	Conserved hypothetical protein
M68	intergenic_spr0645_spr0646	652803	C	T	spr0645	-	spr0646	+	Hypothetical protein	Phospho-beta-gluco or galactosidase truncation
M68	intergenic_spr0659_spr0660	663706	C	T	spr0659	+	spr0660	+	ABC transporter substrate-binding protein	ABC transporter membrane-spanning permease
M13	intergenic_spr0716_spr0717	720291	G	A	spr0716	+	spr0717	+	Cell division regulator negative regulator of FtsZ ring for	Transposase
M19	intergenic_spr0716_spr0717	720041	G	A	spr0716	+	spr0717	+	Cell division regulator negative regulator of FtsZ ring for	Transposase
M27	intergenic_spr0720_spr0721	722207	G	A	spr0720	-	spr0721	-	Hypothetical protein	Conserved hypothetical protein
M37	intergenic_spr0777_sprs03	773246	C	T	spr0777	+	sprs03	+	Conserved hypothetical protein	function down gene not found
M57	intergenic_spr0877_spr0878	867861	C	T	spr0877	+	spr0878	+	Protein-export membrane protein secG	Exoribonuclease R
M37	intergenic_spr0899_spr0900	887235	G	A	spr0899	+	spr0900	+	Degenerate transposase	Hypothetical protein
M27	intergenic_spr0954_spr0955	937836	C	T	spr0954	+	spr0955	+	Hypothetical protein	Hypothetical protein
M24	intergenic_spr0963_spr0964	944610	G	A	spr0963	+	spr0964	+	Hypothetical protein	Hypothetical protein
M18	intergenic_spr0971_spr0972	954770	C	T	spr0971	+	spr0972	+	ABC transporter membrane-spanning permease - macro	Conserved hypothetical protein
M56	intergenic_spr1008_spr1009	991411	G	A	spr1008	-	spr1009	-	Degenerate transposase orf2	Transposase orf1
M55	intergenic_spr1013_spr1014	994467	G	A	spr1013	+	spr1014	+	Conserved hypothetical protein	50S Ribosomal protein L27
M17	intergenic_spr1063_spr1064	1061825	C	T	spr1063	-	spr1064	+	Histidine-containing phosphocarrier protein of the PTS	Glutaredoxin-like protein
M4	intergenic_spr1099_spr1100	1097957	G	T	spr1099	-	spr1100	+	DNA gyrase subunit A	L-lactate dehydrogenase
M14	intergenic_spr1119_spr1120	1119604	CT	C	spr1119	-	spr1120	+	Conserved hypothetical protein	ABC transporter membrane spanning permease - glutamine transport
M21	intergenic_spr1119_spr1120	1119604	CT	C	spr1119	-	spr1120	+	Conserved hypothetical protein	ABC transporter membrane spanning permease - glutamine transport
M29	intergenic_spr1143_spr1144	1145719	G	A	spr1143	-	spr1144	-	Hypothetical protein	DNA processing Smf protein
M49	intergenic_spr1156_spr1157	1160955	G	A	spr1156	-	spr1157	-	Transcriptional attenuation of the pyrimidine operon	endonuclease III DNA repair
M4	intergenic_spr1205_spr1206	1204899	T	G	spr1205	-	spr1206	-	Conserved hypothetical protein	Hypothetical protein
M12	intergenic_spr1205_spr1206	1204899	T	G	spr1205	-	spr1206	-	Conserved hypothetical protein	Hypothetical protein
M23	intergenic_spr1210_spr1211	1207178	C	T	spr1210	-	spr1211	-	Hypothetical protein	50S Ribosomal protein L12
M59	intergenic_spr1293_spr1294	1290016	G	A	spr1293	+	spr1294	-	ABC transporter ATP-binding protein - unknown substrat	Hypothetical protein
M23	intergenic_spr1300_spr1301	1294244	G	A	spr1300	-	spr1301	+	Glutamine amidotransferase	Conserved hypothetical protein
M5	intergenic_spr1333_spr1334	1321266	G	T	spr1333	-	spr1334	-	Peptidoglycan GlcNAc deacetylase	Hypothetical protein
M68	intergenic_spr1410_spr1411	1396707	T	C	spr1410	-	spr1411	+	P-type ATPase - calcium transporter	Conserved hypothetical protein
M11	intergenic_spr1491_spr1492	1469042	AT	A	spr1491	-	spr1492	+	Endopeptidase O	ABC transporter ATP-binding protein - manganese transport
M23	intergenic_spr1495_spr1496	1472309	G	A	spr1495	+	spr1496	-	Thioredoxin-linked thiol peroxidase	Hypothetical protein
M24	intergenic_spr1500_spr1501	1479483	C	T	spr1500	-	spr1501	-	Hypothetical protein	Hypothetical protein
M59	intergenic_spr1522_spr1523	1500023	C	T	spr1522	-	spr1523	-	Hypothetical protein	Hypothetical protein
M16	intergenic_spr1537_spr1538	1519277	G	A	spr1537	-	spr1538	-	Hypothetical protein	Xylan esterase 1
M57	intergenic_spr1537_spr1538	1518923	T	C	spr1537	-	spr1538	-	Hypothetical protein	Xylan esterase 1
M56	intergenic_spr1562_spr1563	1543699	A	G	spr1562	-	spr1563	+	ABC transporter ATP-binding protein - Na%2B export	Degenerate transposase
M29	intergenic_spr1564_spr1565	1545445	G	A	spr1564	+	spr1565	-	Hypothetical protein	Fructokinase
M16	intergenic_spr1581_spr1582	1563533	G	A	spr1581	-	spr1582	-	Primosomal replication factor Y	Hypothetical protein
M29	intergenic_spr1597_spr1598	1575117	G	A	spr1597	-	spr1598	+	Hypothetical protein	Dicarboxylate amino acid:cation Na%2B or H%2B symporter
M30	intergenic_spr1603_spr1604	1579770	G	A	spr1603	+	spr1604	-	Conserved hypothetical protein	Aquaporin Z - water channel protein
M52	intergenic_spr1652_spr1653	1625482	G	A	spr1652	-	spr1653	+	Hypothetical protein	Hypothetical protein
M5	intergenic_spr1668_spr1669	1641845	C	T	spr1668	-	spr1669	+	Galactokinase	GalR member of GalR-LacI family of transcriptional regulators
M55	intergenic_spr1672_spr1673	1645568	C	T	spr1672	-	spr1673	+	Cation diffusion facilitator transporter - heavy metal trans	Conserved hypothetical protein

M18	intergenic_spr1756_spr1757	1724813	G	A	spr1756	-	spr1757	-	DNA-damage-inducible protein	DNA recombination repair
M2	intergenic_spr1829_spr1830	1804130	AT	A	spr1829	-	spr1830	+	Probable nicotinate-nucleotide pyrophosphorylase	Conserved hypothetical protein
M57	intergenic_spr1837_spr1838	1814407	T	TATAAC	spr1837	-	spr1838	-	Alcohol-acetaldehyde dehydrogenase	Hypothetical protein
M61	intergenic_spr1866_spr1867	1837222	CT	C	spr1866	-	spr1867	-	Putative alcohol dehydrogenase	N-acetylglucosamine-6-phosphate deacetylase
M62	intergenic_spr1866_spr1867	1837222	CT	C	spr1866	-	spr1867	-	Putative alcohol dehydrogenase	N-acetylglucosamine-6-phosphate deacetylase
M20	intergenic_spr1886_spr1887	1863935	G	A	spr1886	+	spr1887	-	Degenerate transposase	ABC transporter ATP-binding membrane spanning protein - unknown substrate
M52	intergenic_spr1945_spr1946	1928081	G	A	spr1945	-	spr1946	+	Choline-binding protein	Degenerate transposase orf1
M23	intergenic_spr1948_spr1949	1929846	G	A	spr1948	-	spr1949	-	Hypothetical protein	Hypothetical protein
M59	intergenic_spr1974_spr1975	1965769	C	T	spr1974	+	spr1975	-	Regulator of fucose operon	ABC transporter substrate-binding protein - Zinc Zn2%2B transport
M22	intergenic_spr2017_spr2018	2009951	G	A	spr2017	+	spr2018	-	Degenerate transposase	Degenerate transposase
M43	intergenic_sprt06_spr0778	774375	G	A	sprt06	-	spr0778	+	function up gene not found	Transcriptional repressor of the fructose operon
M37	intergenic_sprt07_spr0918	905160	G	A	sprt07	+	spr0918	+	function up gene not found	Aspartate beta-semialdehyde dehydrogenase
M47	intergenic_sprt07_spr0918	905249	A	T	sprt07	+	spr0918	+	function up gene not found	Aspartate beta-semialdehyde dehydrogenase
M48	intergenic_sprt07_spr0918	905249	A	T	sprt07	+	spr0918	+	function up gene not found	Aspartate beta-semialdehyde dehydrogenase
M32	intergenic_sprt11_spr1617	1590749	C	T	sprt11	+	spr1617	-	function up gene not found	Sucrose-6-phosphate hydrolase
